# Are platelet concentrate scaffolds superior to traditional blood clot scaffolds in regeneration therapy of necrotic immature permanent teeth? A systematic review and meta-analysis

**DOI:** 10.1186/s12903-022-02605-4

**Published:** 2022-12-09

**Authors:** Qianwei Tang, Hua Jin, Song Lin, Long Ma, Tingyu Tian, Xiurong Qin

**Affiliations:** 1 College of Stomatology, Hospital of Stomatology, Guangxi Medical University, Nanning, Guagnxi China; 2 Department of Stomatology, Harbin Institute of Technology Hospital, Harbin, HeiLongjiang China; 3 Department of Pediatric Dentistry, Jinan Stomatology Hospital, Jinan, 250001 Shandong Province China

**Keywords:** Platelet concentrates, Regenerative endodontics, Platelet-rich plasma, Platelet-rich fibrin, Blood clot, Immature tooth

## Abstract

**Background:**

The effectiveness of platelet concentrates in promoting root development of necrotic immature permanent teeth is unclear. The present study evaluated whether the platelet concentrate protocol was superior to the traditional blood clot protocol in regeneration therapy.

**Methods:**

We searched Electronic databases, such as PubMed, Cochrane Library, ClinicalTrials and EMBASE. Randomized controlled trial studies, cohort studies, case-control studies and cross-sectional studies were included, in which platelet-rich concentrates were tested for periapical healing and root development, with the blood clot treatment protocol as the control group. Clinical and radiographic outcomes were considered. Selected articles were assessed for risk of bias. Pooled risk ratios (risk ratio, RR) were calculated for clinical success, responses to cold and electric pulp tests, periapical lesions, apex closure, root lengthening, and thickening of the dentin walls. Subgroup meta-analysis were conducted according to the type of platelet concentrate used.

**Results:**

Of the 1272 screened studies, 13 randomized controlled studies, 2 case-control studies and 1 cohort study were selected, in which 465 immature necrotic permanent teeth, particularly incisors and premolars, were treated. Of these 465 teeth, 457 (98.2%) in both the control and experimental groups remained clinically asymptomatic for the entire study duration, whereas eight (1.8%) showed signs and symptoms of failure, including spontaneous pain, sensitivity to percussion or reinfection. Compared with control teeth, teeth treated with PRP achieved better apical healing than BC group (RR 1.13, 95% CI 1.01–1.26, *P* = 0.03), and teeth treated with platelet concentrates showed improved apical closure (RR 1.04, 95% CI 0.86–1.25, *P* = 0.69), root lengthening (RR 1.01, 95% CI 0.74–1.39, *P* = 0.93), and thickening of the dentin walls (RR 1.35, 95% CI 0.95–1.93, *P* = 0.09), although these differences were not statistically significant.

**Conclusions:**

Platelet concentrates can be used as successful scaffolds for regenerative endodontic treatment of necrotic immature permanent teeth, and PRP as a scaffold may achieve better periapical healing of teeth with periapical inflammation, although they did not differ significantly from conventional blood clot scaffolds in development of the root.

**Supplementary Information:**

The online version contains supplementary material available at 10.1186/s12903-022-02605-4.

## Background

Pulp necrosis in an immature permanent tooth with an open apex is challenging to treat, as the open apex can limit root canal disinfection and obturation. Although these teeth are conventionally managed by apexification with calcium hydroxide(Ca(OH)_2_) [1] or mineral trioxide aggregate (MTA) [2, 3], neither procedure allows for thickening of the root wall or continued development of the root [4, 5]. Revascularization of an immature, non-vital tooth was found to increase root length and dentin wall thickness [6], with many case reports and clinical studies showing that revascularization resulted in favorable outcomes [7].

Regenerative endodontics (RE) are based on three core principles for tissue engineering: appropriate sources of stem/progenitor cells, growth factors that promote stem cell differentiation, and a three-dimensional (3D) physical scaffold that can sustain cell growth and differentiation [8, 9]. The main sources of stem cells are in the apical papilla [10] and periapical tissues of immature permanent teeth [11]. Growth factors are normally secreted by platelets and other cells present in blood clots [7]. In most cases, the scaffolds are naturally provided by intracanal blood clots, dentin walls and fibrin mesh provided by platelets in the coagulum [7, 9].

Regarding the scaffold, the classic treatment protocol of dental pulp revascularization consists of piercing the apex and to filling the root canal with a blood clot. This blood clot may act as a scaffold supporting angiogenesis, providing a pathway for the migration of stem cells from the periapical area. In clinical practice, however, dentists cannot always induce sufficient blood to serve as a scaffold, as the clot may be influenced by Ca(OH)_2_ root canal disinfectants and local anesthetics containing a vasoconstrictor such as adrenaline. In addition, the content of growth factors in blood clots is limited and unpredictable. Many recent studies have investigated the use of platelet concentrate scaffolds in revascularization protocols [12–18]. Compared with blood clots, platelet concentrates have shown many advantages, including the ability to stabilize blood clots, maintain growth factor levels, and promote tissue regeneration [19–23]. Platelet-rich plasma (PRP) and platelet-rich fibrin (PRF) are two concentrated sources of platelets widely used in RE. PRP, which contains leukocytes and high platelet concentrations, ranging from 160 to 740% compared with whole blood [24], can be prepared from anticoagulated blood by double centrifugation and requires an activator before use [25]. PRF is a second-generation platelet concentrate that does not require biochemical handling of blood and is easy to procure [26]. PRF can be prepared from non-anticoagulated blood using a single centrifugation step [27] and does not require an activator before use. Depending on the method of preparation, retention time, transfer process, fibrin structure, platelets and released cell growth factors, PRF derivatives can include pure PRF (P-PRF), leukocyte- and platelet-rich fibrin (L-PRF), advanced PRF (A-PRF), and injectable PRF (i-PRF).

Increasing the concentration of platelets in these preparations increases the number and concentrations of growth factors secreted by these platelets. These growth factors stimulate the proliferation of stem cells, inducing tissue healing and regeneration. Theoretically, PRP and PRF stimulate stem cell proliferation and increase the expression of osteoprotegerin proteins and alkaline phosphatases, thus hastening the revascularization of young permanent teeth with pulp necrosis [28]. To date, however, few studies have assessed these effects, limiting the widespread use of these treatment protocols in clinical practice. The present meta-analysis was designed to determine the effectiveness of platelet concentrates in the treatment of immature necrotic teeth. The main objectives of this study were (a) to radiographically evaluate the resolution of periapical lesions, apex closure, root lengthening and thickening of the root canal walls; and (b) to clinically evaluate the symptoms and responses to pulp sensibility testing after treatment.

## Materials and methods

### Study protocol and research question

This study was not registered in the International Prospective Register of Systematic Reviews (PROSPERO), but conducted in accordance with the Preferred Reporting Items for Systematic Reviews and Meta-Analysis (PRISMA) 2020 guidelines [29]. The research question of this review was based on the PICOS framework: Population (P): immature necrotic teeth; Intervention (I): two different scaffolds used for blood clots and platelet concentrates in pulp revascularization; Comparisons (C): compare the efficacy of two different scaffolds for blood clots and platelets; Outcome (O): the clinical symptoms, pulp vitality and imaging findings(periapical healing, root lengthening, root canal thickening, and apical closure) detected during follow-up; Study Design (S): Randomized controlled trial studies (RCT), prospective or retrospective cohort studies, case-control studies and cross-sectional studies were retrieved for inclusion.

### Eligibility criteria

Studies were included if the studies investigating the effects of platelet concentrates and blood clots on the revascularization of immature teeth with necrotic pulp in humans. Reviews, abstracts, case reports and series, comments, letters to editor conference proceedings, in vitro investigations, and animal studies were excluded.

### Literature search strategy and selection of papers

The PubMed, Cochrane, ClinicalTrials and EMBASE databases were searched for studies published in English through April 2022 that compared blood clots and platelet-rich concentrates in the regeneration of necrotic immature permanent teeth in humans. The search terms included “regenerat* OR revital* OR revasculari*”, “pulp necrosis OR necrosis of the pulp OR necrotic pulp OR pulpal necrosis OR non vital* OR pulp infection* OR infected pulp* OR pulpal infection*", "Immature permanent tooth OR immature tooth OR open apex OR open apices", "platelet concentrate", "platelet-rich plasma OR PRP", "platelet-rich fibrin OR PRF", and "concentrated growth factor OR CGF". The search terms were used alone or in combination using the Boolean operators OR and AND (Table 1). The references of all selected publications were manually searched to identify additional studies that met the inclusion criteria.

The searches were imported into the EndNote version 20 library. Duplications were identified and removed. Titles and abstracts of each retrieved record were screened to exclude any papers not fulfilling inclusion criteria. Two independent reviewers (S Lin and L Ma) identified the studies extracted from the searches. When there was uncertainty on the eligibility of an article, the study was adjudicated based on the discussion and consensus between the two reviewers and a third reviewer (XR Qin).

### Data extraction

Relevant data of included papers were independently extracted in duplicate by two authors (QW Tang and H Jin). Data extracted from the selected studies included study information (author, year, and study design), patient information (age, sex, tooth type, and number of patients), diagnostic information (pulp and periapical status of treated teeth and etiology of pulp necrosis), treatment protocols (instrumentation for root canals, irrigation and intracanal medications), and follow-up evaluations (pulp response, periapical healing, root lengthening, root canal thickening, and apical closure). To standardize the scores utilized to evaluate study results, which were usually scored as excellent, good, satisfactory, and unsatisfactory, and to perform proper statistical analyses, the outcomes were dichotomized, with results scored as excellent and good being aggregated. To avoid the risk of retrieval bias, the authors were not contacted about missing information required for the meta-analysis.

### Quality assessment

The quality assessment was carried out independently by two authors (QW Tang and H Jin) and any disagreements were resolved by consensus. The quality of the included RCT researchs was assessed using the Cochrane risk of bias (RoB) tool, including the following six aspects: ① Generation of random sequence; ② Hiding of random sequence; ③ Whether blind method is adopted; ④ Integrity of settlement data; ⑤ Whether the research results are selectively reported; ⑥ Other sources of bias. The cohort study and case-control study were evaluated with the Newcastle Ottawa Scale (NOS) (https://www.ohri.ca//programs/clinical_epidemiology/oxford.asp). The NOS evaluates the methodological quality of individual studies following a star system based on 8 domains grouped into 3 main domains: patient selection, comparability of study groups, and outcome assessment. Cohort and case-control studies may receive up to 9 stars. Studies were categorized as high-quality, moderate quality and low quality if they reached 7–9 (cohort and case-control studies), 4–6 stars and 0–3 stars, respectively.

### Statistical analysis

Kappa test was used for quality assessment of article identification, screening, data extraction and quality assessment to evaluate the agreement among reviewers using SPSS 20.0 software (SPSS, Inc., Chicago, IL, USA).

The review manager 5 software(Revman5.4) was used to analyze the combined effect, heterogeneity and publication bias. To determine the effects of platelet concentrates as canal scaffolds, pooled risk ratios (RRs) were calculated for clinical success, responses to cold and electric pulp tests, periapical lesions, apex closure, root lengthening, and thickening of the dentin walls. Heterogeneity was assessed using I^2^ statistics and Cochrane’s Q test, with I^2^ > 50% or *P* < 0.10 on Cochrane’s Q test indicating substantial heterogeneity [30]. *P* values < 0.05 were defined as statistically significant.

If five or more studies are included, publication bias was evaluated by visual inspection of the funnel plot [31].The CMA3.0 software was used to analyze the Bgger’s and Egg’s test and sensitivity analyses. Sensitivity analyses (one study removed) were used to evaluate the stability of the results of the included studies.

## Results

### Systematic search

**Fig. 1:**
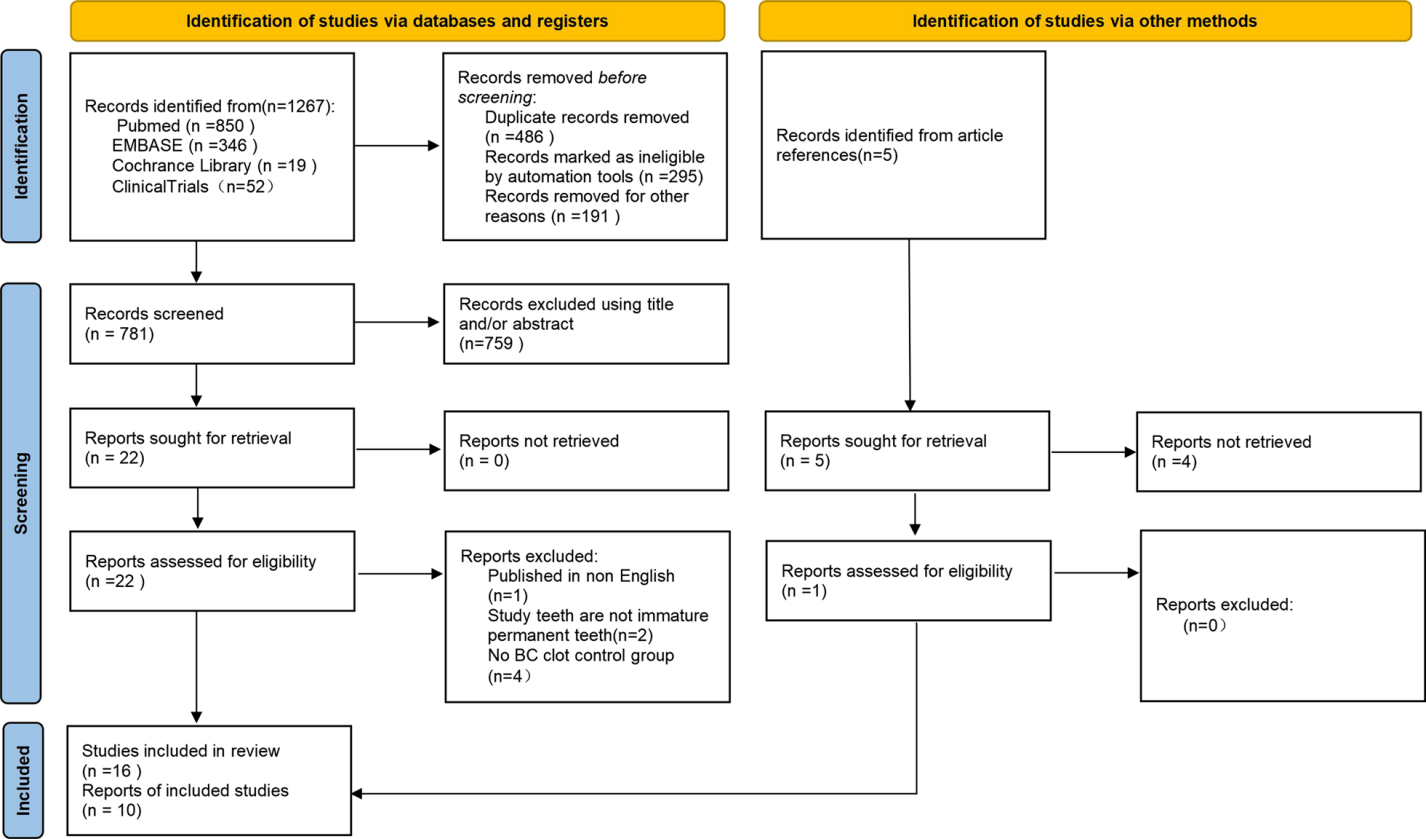
The PRISMA flow diagram summarizing study selection process

The primary search resulted in 1272 papers published between 2012 and 2022; of these, 23 met the initial inclusion criteria. Reading of complete texts resulted in the inclusion of 16 studies, 11 RCT parallel studies [12, 14, 15, 32–39] two RCT split-mouth studies [16, 40], two case-control studies [41, 42] and one cohort study [43] (Fig. 1). Seven studies were excluded because there were published in non English [44], the included teeth were not immature permanent teeth [17, 45], and there was no blood clot in the control group [18, 46–48] (Additional file 1). Details on the selection process of articles were represented with a flow diagram (Fig. 1).

A total of 465 immature necrotic teeth, particularly incisors and premolars, were treated (Tables  2). Etiologically, pulpal necrosis was most frequently secondary to caries, trauma or developmental anomalies (dens envaginatus or dens invaginatus). Of these 465 teeth, 130 were treated with adjunctive PRP; 120 with an adjunctive platelet concentrate scaffold; including 79 with PRF, 11 with L-PRF, and 30 with concentrated growth factor (CGF) scaffolds; and 215 with a blood clot alone. The treatment period was 12 months in 11 articles [12, 16, 32–38, 40, 41], 18 months in two articles [14, 15] and 36 months in one article [43]. Other studies recorded postoperative readings until teeth achieved complete healing, with treatment periods ranging from 10–49 months in one study [39] and 6–69 months in the other [42]. Twenty-four teeth, including 11 treated with blood clots, six treated with PRP, 5 treated with PRF, and two treated with L-PRF, dropped out in four studies because of no or irregular follow-up [16, 33, 39, 43]. Details of the operative protocols in each included study are shown in Table 2.

**Table 1:** The search strategy

Database	Key words	Results
Pubmed	#1 Pulp necrosis OR necrosis of the pulp OR Necrotic pulp OR Pulpal necrosis OR Non vital* OR Pulp infection* OR Infected pulp* OR Pulpal infection*	850
	#2 Regenerat* OR Revital* OR Revasculari*	
	#3 Immature permanent tooth OR immature tooth OR open apex OR open apices	
	#4 platelet concentrate OR platelet-rich plasma OR PRP OR platelet-rich fibrin OR PRF OR concentrated growth factor OR CGF	
	#3 AND #4 (138); #1 AND #4 (401); #2 AND #3 AND #4 (104); #1 AND #3 AND #4 (59); #1 AND #2 AND #4(96); #1 AND #2 AND #3AND #4 (52)	
	Last update posted on or before 04/30/2022	
Clinicaltrials	Pulp necrosis OR necrosis of the pulp OR Necrotic pulp OR pulpal necrosis OR Non vital* OR Pulp infection* OR Infected pulp* OR Pulpal infection* | Regenerat* OR Revital* OR Revasculari* | Last update posted on or before 04/30/2022 (36)	52
	Pulp necrosis OR necrosis of the pulp OR Necrotic pulp OR Pulpal necrosis OR Non vital* OR Pulp infection* OR Infected pulp* OR Pulpal infection* | platelet concentrate OR platelet-rich plasma OR PRP OR platelet-rich fibrin OR PRF OR concentrated growth factor OR CGF | Last update posted on or before 04/30/2022 (11)	
	Immature permanent tooth OR immature tooth OR open apex OR open apices | platelet concentrate OR platelet-rich plasma OR PRP OR platelet-rich fibrin OR PRF OR concentrated growth factor OR CGF | Last update posted on or before 04/30/2022 5	
	Regenerat* OR Revital* OR Revasculari* | platelet concentrate OR platelet-rich plasma OR PRP OR platelet-rich fibrin OR PRF OR concentrated growth factor OR CGF | Last update posted on or before 04/30/2022 (0)	
Cochrane libray	Pulp necrosis OR necrosis of the pulp OR Necrotic pulp OR Pulpal necrosis OR Non vital* OR Pulp infection* OR Infected pulp* OR Pulpal infection* in Title Abstract Keyword AND Regenerat* OR Revital* OR Revasculari* in Title Abstract Keyword AND Immature permanent tooth OR immature tooth OR open apex OR open apices in Title Abstract Keyword AND (platelet concentrate) OR (platelet-rich plasma OR PRP) OR (platelet-rich fibrin OR PRF)OR (concentrated growth factor OR CGF) in Title Abstract Keyword—(Word variations have been searched)	19
	Last update posted on or before 04/30/2022	
Embase	#1 Pulp necrosis OR necrosis of the pulp OR Necrotic pulp OR Pulpal necrosis OR Non vital* OR Pulp infection* OR Infected pulp* OR Pulpal infection*	346
	#2 Regenerat* OR Revital* OR Revasculari*	
	#3 Immature permanent tooth OR immature tooth OR open apex OR open apices	
	#4 platelet concentrate OR platelet-rich plasma OR PRP OR platelet-rich fibrin OR PRF OR concentrated growth factor OR CGF	
	#1 AND #2 AND #3 AND #4 (0); #1 AND #2 AND #4 (0); #2 AND #3 AND #4 (0); #1 AND #4 (1); #1 AND #2 (319);#1 AND #3 (6); #2 AND #3 (20)	
	Last update posted on or before 04/30/2022

**Table 2:** Patients, teeth, pretreatment signs and symptoms, and treatment information of the included studies

Author	Study design	Patients, sex (age range)	Tooth type	Etiology of pulp necrosis	Groups of Study(n)	Teeth dropout (n)	Follow-up Time	Pretreatment signs and symptoms		Treatment of the root			Local
							(months)	Preoperative signs and symptoms (n)	Periapical radiolucid lesion (n)	Instrumentation	Irrigation	Intracanal medication	Anesthetics
Jadhav et al. (2012)[12]	RCT, parallel	12 M/8 F	Incisors	NR	BC (10)	0	12	NR	Yes or No	Minimal	2.5% NaOCl	TAP	Without adrenaline
		(15–28 years)			BC + PRP (10)	0						(MET, CF, MIN,)	
Bezgin et al. (2015)[14]	RCT, parallel	10 M/8 F (7–12 years)	Premolars incisors	Caries Trauma	BC (10)	0	18	Physiologic (4)	No (4)	No	2.5% NaOCl Saline 0.12% CHX 5% EDTA	TAP for 3 wk (CF, MET, CR)	Without adrenaline
					PRP (10)	0		Sensitive (16)	Yes (16)				
Narang et al. (2015)[15]	RCT, parallel	15 patients (< 20 years)	NR	NR	BC (5)	0	18	NR	Yes (15)	Minimal	2.5% NaOCl	TAP for 4 wk	NR
					PRP + Collagen (5)	0							
					PRF (5)	0							
Sharma et al	RCT, parallel	16 patients	Incisors	Trauma	BC (4)	0	12	Spontaneous pain (9)	No (3)	Minimal	2.5% NaOCl	TAP for 4 wk	without adrenaline
(2016)[32]		(10–25 years)			PRF (4)	0		Discoloration (7)	Yes (13)				
					BC + Collagen (4)	0		Sinus (2) Percussion (2)					
					BC + PLGA (4)	0							
Alagl et al. (2017)[16]	RCT, split mouth	9 M/6 F	Premolars	Caries	BC (15)	0	12	Physiologic (4)	No (5)	No	1.5% NaOCl	TAP for 3 wk (MET, CF, MIN,)	NR
		(7–12 years)	incisors	Trauma	PRP (15)	0		Sensitive (26)	Yes (25)		2.5% NaOCl		
											0.12% CHX		
Shivashanker et al. (2017)[33]	RCT, parallel	60 patients (6–28 years)	NR	Caries	BC(20)	5	12	NR	NR	Minimal	5.25% NaOCl	TAP for 3 wk (MET 400 mg, CF 200 mg, MIN 100 mg)	NR
				Trauma	PRP(20)	1							
					PRF(20)	0							
Lv et al. (2018)[41]	Case–control	5 M/5 F	Premolars	DE	BC (5)	0	12	NR	Yes (10)	No	1% NaOCl	4 wk (ciprofloxacin, metronidazole, cefaclor (1:1:1))	2% lidocaine without
		(9–14 years)	Incisors	Trauma	PRF (5)	0					Saline		adrenaline
Mittal et al. (2019)[34]	RCT parallel	NR	Incisors	NR	BC(4)	0	12	NR	NR	Minimal	2.5% NaOCl	4 wk (Double Antibiotic Paste)	NR
					BC + PRF (4)	0							
Ragab et al. (2019)[35]	RCT, parallel	15 M/7 F	Incisors	Trauma	BC(11)	0	12	abscess	Yes (22)	No	5% NaOCl	3 wk (metronidazole ciprofloxacin 1: 1)	3%
		(7–12 years)			BC + PRF (11)	0		fistula discoloration			saline		mepivacaine
Ulusoy et al. (2019)[39]	RCT, parallel	44 M/33 F	Incisors	Trauma	BC (22)	1	Until complete	NR	Yes or No	No	1.25% NaOCl	TAP for 4 wk	2% mepivacaine
		(8–11 years)			PRP (22)	4	healing 10–49				2% CHX		
					PRF (22)	5					17% EDTA		
					PP(22)	5							
ElSheshtawy et al. (2020)[36]	RCT, parallel	15 M/11 F (> 7;12.66 ± 4.77)	Incisors	DE	PRP (14)	1	12	Sensitive (14)	No (13)	Minimal	5.25% NaOCl	TAP until resolution of clinical signs and symptoms of infection	without vasoconstrictor
				Trauma	BC (17)	0		Physiologic(17)	Yes (18)		17% EDTA		
Uppala et al. (2020)[38]	RCT, parallel	10 M/14 F	Incisors	NR	PRF(8)	0	12	NR	Yes (24)	Minimal	NR	TAP for 4 wk	NR
		(13–24 years)			BC(8)	0							
					BC + collagen (8)	0							
Rizk et al. (2020)[40]	RCT, split mouth	7 M/6 F	Incisors	Caries	PRP (13)	0	12	NR	Yes or No	Minimal	2% NaOCl	TAP for 3wk	NR
		(8–14 years)		Trauma	BC + collagen (13)	0					17% EDTA		
Ramachandran et al. (2020)[37]	RCT, parallel	27 M/13F	NR	NR	BC + PRP + collagen (20)	16	12	NR	Yes (40)	Removal of necrotic tissue	5.25% NaOCl	TAP for 3 wk	NR
		(15–54 years)			BC (20)						1% NaOCl		
											sterile water		
											17% EDTA		
Meschi et al. (2021)[43]	Cohort study	27 patients	Premolars	DE, DI	BC (18)	4	36	No (5)	No (9)	NR	1.5% NaOCl	Double antibiotic paste (metronid ciprofloxacin)	local anesthesia with adrenaline
		(6–25 years)	Incisors	Trauma Caries	BC + L-PRF (11)	2		Yes (24)	Yes (20)		Saline	calcium hydroxide	
								(Swelling,			17% EDTA		
								sinus tract,					
								percussion, pain,					
								discoloration)					
Cheng et al. (2022)[42]	Case–control	34 M/ 28F	Incisors	Trauma	BC (32)	0	Jun-69	Swelling or sinus tract (21)	Yes or No	No or minimal	0.5–1.5% NaOCl saline	TAP or calcium hydroxide for 2 wk	NR
		(8.6 ± 1.4 years)			CGF (30)	0		Mobility (31)			17% EDTA		

M: male; F: female; NR: not reported; PRP: platelet-rich plasma; PRF: platelet-rich fibrin; BC: blood clot; NaOCl: sodium hypochlorite; TAP: triple antibiotic paste (metronidazole, ciprofloxacin, minocycline, 1:1:1); RCT: randomized clinical trial; EDTA: ethylendiamine-tetra-acetate; CHX: chlorhexidine; PLGA: poly-lactic-co-glycolic acid; DE, dens envaginatus; DI, dens invaginatus; CGF, concentrated growth factor

### Quality assessment and Kappa’s test

**Fig. 2:**
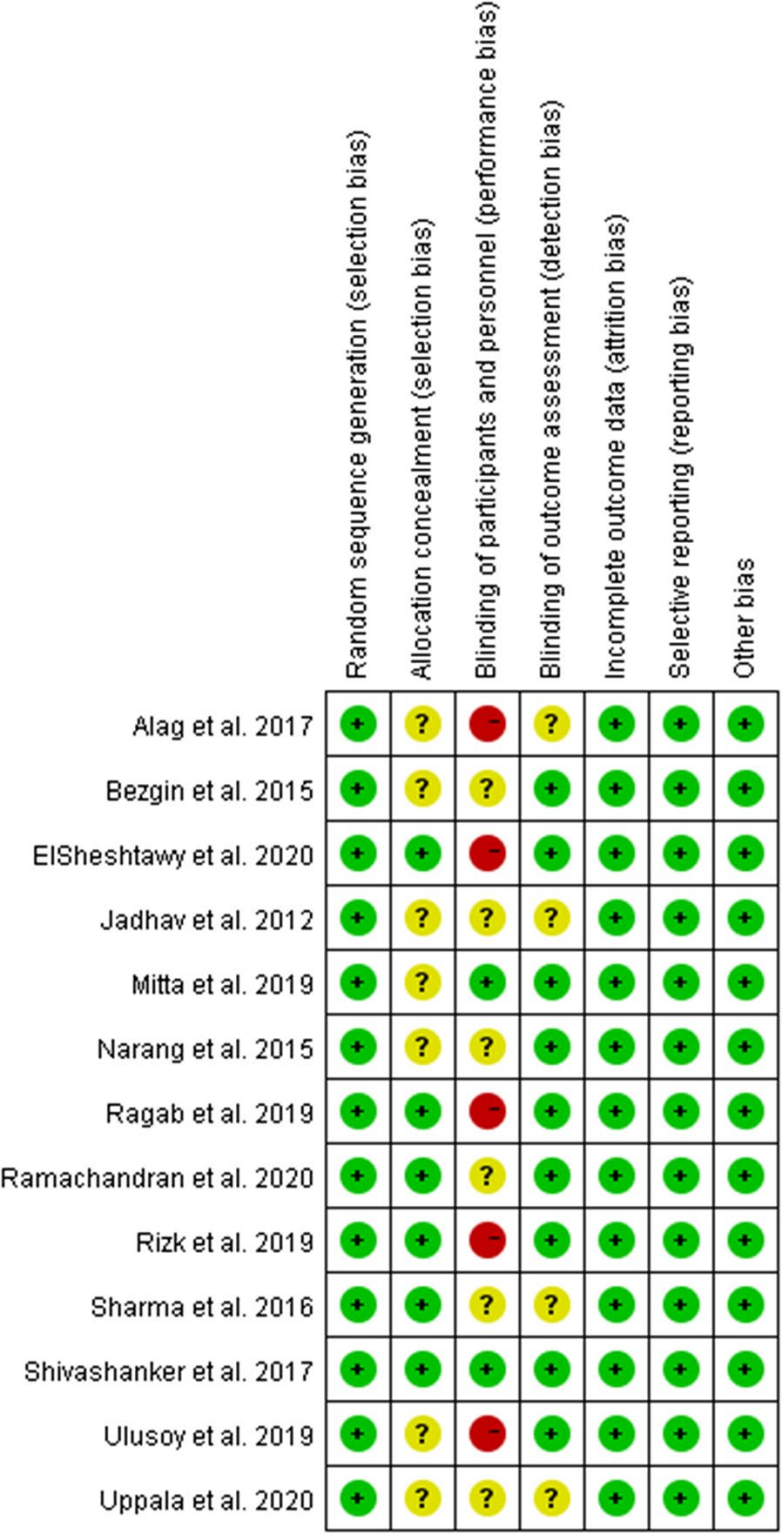
Risk of bias summary showing estimates of the risk of bias items of the included studies

**Fig. 3:**
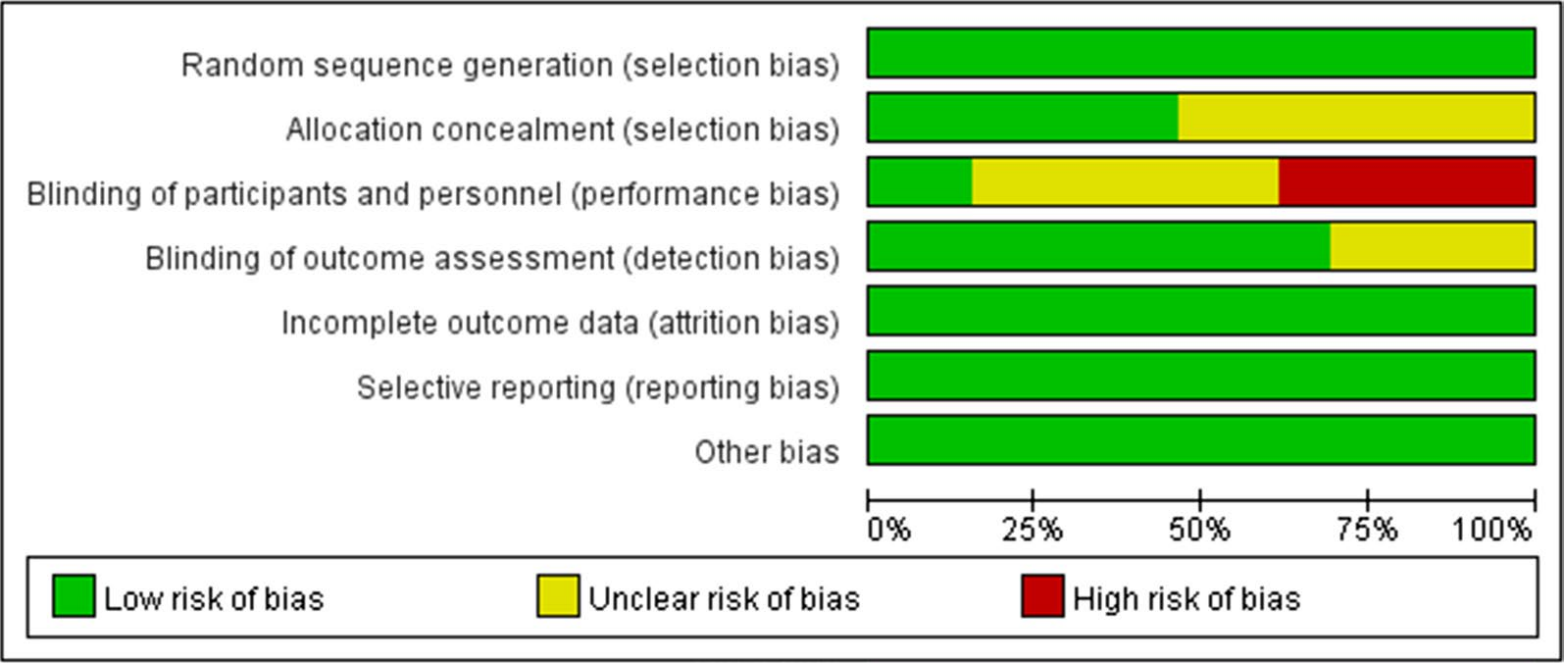
Risk of bias graph showing estimates of each risk of bias item presented as percentages across all included studies

According to the different design types of included articles, different methods for evaluating the grade of articles are selected. The risk of bias assessment on included studies were available in Figs. 2, 3, Tables 3 and 4. Overall, only four studies [33, 41–43] were considered to have a low risk of bias (RoB) or high-quality, whereas seven [12, 14, 15, 32, 34, 37, 38] were considered to have unclear risk of RoBs and five [16, 35, 36, 39, 40] were considered to have high risk of RoBs, respectively.

**Table 3:** Quality assessment according to Newcastle–Ottawa of the included case–control studies

items		Lv et al. (2018)	Cheng et al. (2022)
Selection	Is the case definition adequate?	★	★
	Representativeness of the cases	★	★
	Selection of controls		★
	Definition of controls	★	★
Comparability	Comparability of cases and controls on the basis of the design or analysis	★★	★★
Exposure	Ascertainment of exposure	★	★
	Same method of ascertainment for cases and controls	★	★
	Non-response rate	★	★
Grade	9★	8★	9★

**Table 4:** Quality assessment according to Newcastle–Ottawa of the included cohort studies

		Meschi et al. (2021)
Selection	Representativeness of the exposed cohort	★
	Selection of the non-exposed cohort	★
	Ascertainment of exposure	★
	Demonstration that outcome of interest was not present at start of study	★
Comparability	Comparability of cohorts on the basis of the design or analysis	
Outcome	Assessment of outcome	★
	Was follow-up long enough for outcomes to occur	★
	Adequacy of follow up of cohorts	★
Grade	9★	***7***★

Kappa coefficients of reviewers in article identification and screening, data extraction and quality assessment were 0.894, 0.897, 1.000 respectively (Additional file 2). All are greater than 0.81, indicating strong agreement among reviewers [49].

### Characteristics of the clinical protocol

In general, the clinical protocol in the included articles consisted of the removal of the necrotic pulp and irrigation with NaOCl or EDTA, with minimal or no use of mechanical instrumentation on the dentin walls (Table 2). The canals were subsequently treated with double or triple antibiotic paste or calcium hydroxide for 3 or 4 weeks. Following the disappearance of tooth symptoms, platelet concentrate was injected into the root canal, where it polymerized in gel form. Platelet concentrate was used alone [14, 16, 33, 36, 39, 41, 42], in combination with a blood clot [12, 34, 35], or in conjunction with a collagen sponge and blood clot [15, 32, 37, 38, 40, 43]. A bio-ceramic material (MTA or iRoot BP Plus), glass ionomer cement or bonded resin restoration was subsequently inserted into the root canal over the platelet concentrate or blood clot.

The protocols used for producing PRP were very similar among the articles, with all using the same anticoagulant and two centrifugation steps at the same speed. Activators, however, differed from studies using 10% calcium chloride alone or with sterile bovine thrombin (100 U/mL). One study did not report the details of making PRF [15], whereas, in another study [33], PRF was prepared from nonanticoagulated blood using a single centrifugation step, with this preparation not requiring an activator before use [20].

### Radiographic evaluation and imaging analysis

Radiographic evaluation included both qualitative and quantitative findings. Imaging techniques included 3D imaging, such as cone-beam computed tomography (CBCT), and 2D imaging, such as periapical digital radiography. Quantitative analyses were performed using Image J software. The indicators measured in different studies are also different. The indicators measured included the perimeter of periapical radiolucency [35], the periapical lesion area, and the total radiographic root area (RRA) [37, 42, 43]. The length of the root canal was also measured, although these indices also differed.

### Characteristics of studies included in systematic evaluations

Although articles measuring qualitative parameters could be merged and included in a meta-analysis [12, 14–16, 32–34, 38, 39, 41], articles measuring quantitative parameters could not be merged and included in systematic evaluation [35–37, 40, 42, 43]. The clinical and radiographic outcomes of the treatment and control groups in these articles are summarized in Tables 2 and 5.

**Table 5:** Clinical and radiographic outcomes of test and control groups of studies included in the systematic evaluation but not in the meta-analysis

Author (year)	No. of teeth (group)	Teeth dropout (n)	Follow-up (month)	Clinical success (n)	Response to cold and electric pulp test (n)	Radiographic assessment and standardization	Results
Ragab et al. (2019)[35]	BC (11)	0	12	NR	NR	2D radiographs	Both groups showed radiographic evidence of periapical healing and calcific bridges either cervical and /or apical
	BC + PRF (11)	0				Image J software	Treatment outcomes did not differ significantly between BC and PRF scaffold
ElSheshtawy et al. (2020)[36]	PRP (14)	1	18	No (12)	Positive (0), Negative (13)	2D radiographs	Changes in RL*, RDT**, AFW***, RRA^ and PAD^^ over time significant in both groups
	BC (17)	0		No (16)	Positive (0), Negative (17)	CBCT	No difference between the PRP and BC groups, using both radiographic and CBCT methods
						Image J	
Rizk et al. (2020)[40]	PRP (13)	0	12	No (13)	Positive (0), Negative (13)	2D radiographs	Compared with BC, PRP-treated teeth showed statistically significant increases in radiographic root length, width, and periapical bone density, and a decrease in apical diameter
	BC + collagen (13)	0		No (13)	Positive (0), Negative (13)	Image J software	
Ramachandran et al. (2020)[37]	BC + PRP(20)	16	06-Dec	No (10)	Positive (0), Negative (10)	2D radiographs	No difference in the percentage changes in RRA^ between the BC and BC + PRP groups
	BC (20)			No (14)	Positive (0), Negative (14)	Image J software	
Meschi et al. (2021)[43]	BC (18)	4	36	No (14)	Positive (4), Negative (10)	2D radiographs	Volume root hard tissue thickness and apical area significantly better in the BC than in the BC + L-PRF group
	BC + LPRF (11)	2		No (9)	Positive (5), Negative (4)	CBCT	No significant differences in root length and maximum root hard tissue thickness between the BC and LPRF groups
						Image J software	
Cheng et al. (2022)[42]	BC (32)	0	Jun-69	50 together#	NR	2D radiographs	Scaffold was a significant predictor of success; BC had a significantly reduced risk for failure than CGF, the induced bleeding technique appeared more appropriate for the management of traumatized teeth with REPs
	CGF (30)	0				Image J software	

^#^In the article, the author did not mention the number of successful people in BC and CGF groups, but only provided the total number of successful people；*: RL: root length; **:RDT: root dentinal thickness; ***: AFW: pical foramen width; ^: RRA: radiographic root area; ^^: periapical area diameter

Of the six articles included in Table 5, four found that scaffolds formed by platelet concentrates and by blood clots achieved better therapeutic effects, but the outcomes in these two groups did not differ significantly. One study found that radiographic root length, width, and periapical bone density were significantly higher and apical diameter significantly lower in PRP-treated teeth than in BC-treated teeth [40], whereas another study found that the risk of failure was significantly lower with BC than CGF, suggesting that the induced bleeding technique appears to be more appropriate for the management of traumatized teeth with REPs [42].

One study found that two patients in the BC group and one in the PRP group presented with signs of reinfection at the second recall appointment after 6 months [36], whereas another article described four patients with pain, but their treatment was not reported [37].

### Synthesis of results (meta-analysis)

A meta-analysis compared the effectiveness of platelet concentrate and BC scaffolds for the treatment of young, immature, necrotic, permanent teeth (Table 6). Forest plots were generated for clinical success, pulp response, periapical healing, root lengthening, root canal thickening and apical closure, and subgroup analyses were performed based on the type of platelet concentrate used (i.e., PRP or PRF).

**Table 6:** Clinical and radiographic outcomes of treatment and control groups after 12–18 months (meta)

Author (year)	No. of teeth (group)	No. of teeth drop out	Outcomes									
			Clinical success (n)	Response to cold and electric pulp test (n)	Periapical healing		Apex closure		Root lengthening		Root canal thickening	
					Excellent + Good	Satisfactory (unsuccessful)	Excellent + Good	Satisfactory (no change)	Excellent + Good	Satisfactory (no change)	Excellent + Good	Satisfactory (no change)
Jadhav et al. (2012)[12]	10 (PRP)	0	No (10)	NR	9	1	10	0	9	1	8	2
	10 (BC)	0	No (10)	NR	7	3	5	5	6	4	3	7
Bezgin et al. (2015)[14]	10 (PRP)	0	No (10)	Positive (5), Negative (5)	7**	0	7	3	&	&	&	&
	10 (BC)	0	No (10)	Positive (2), Negative (8)	8**	0 (1)	6	4				
Narang et al. (2015)[15]	5 (PRP)	0	No (5)	NR	4	1	3	2	2	3	1	4
	5 (PRF)	0	No (5)		5	0	2	3	5	0	5	0
	5 (BC)	0	No (5)		3	2	3	2	2	3	2	3
Sharma et al. (2016)[32]	4 (BC)	0	No (4)	NR	3	1	3	1	4	0	2	2
	4 (PRF)	0	No (4)		4	0	4	0	4	0	3	1
	4 (Collagen)	0	No (4)		4	0	3	1	4	0	3	1
	4 (PLGA)	0	No (4)		1	3	2	2	4	0	1	3
Alag et al. (2017)[16]	15 (PRP)	0	No (15)	Positive (13), Negative (2)	12***	0	14	1	#	#	#	#
	15 (BC)	0	No (15)	Positive (6), Negative (9)	13***	0	8	7				
Shivashanker et al. (2017)[33]	20 (PRF)	0	No (20)	Positive (3), Negative (17)	15	3 (2)	4	10 (6)	6	7 (7)	6	8 (6)
	20 (PRP)	1	No (19)	Positive (3), Negative (16)	19	0	4	12 (3)	5	9 (5)	5	11 (3)
	20 (BC)	5	No (15)	Positive (2), Negative (13)	12	3	5	8 (2)	4	9 (2)	3	11 (1)
Lv et al. (2018)	5 (PRF)	0	No (5)	Positive (3), Negative (2)	5	0	4	1	4	1	4	1
[41]	5 (BC)	0	No (5)	Positive (1), Negative (4)	5	0	4	1	4	1	4	1
Mittal et al. (2019)[34]	4 (PRF)	0	No (4)	NR	3	1	3	1	0	4	4	0
	4 (BC + collagen)	0	No (4)	NR	4	0	1	3	1	3	3	1
Ulusoy et al. (2019)[39]	PRP (18)	4	No (18)	Positive (13), Negative (5)	18	0	10	2 (6)	&	&	&	&
	BC (21)	1	No (20)	Positive (20), Negative (1)	20	1	15	1 (5)				
	PRF (17)	5	No (16)	Positive (16), Negative (2)	16	1	9	5 (3)				
	PP (17)	5	No (17)	Positive (16), Negative (2)	17	0	7	8 (2)				
Uppala et al. (2020)[38]	PRF (8)	0	No (8)	NR	8	0	8	0	1	7	2	6
	BC (8)	0	No (8)	NR	6	2	8	0	6	2	4	4
	BC + collagen (8)	0	No (8)	NR	8	0	5	3	3	5	2	6

NR: not reported; PRP: platelet-rich plasma; PRF: platelet-rich fibrin; BC: blood clot

^**^One tooth in the BC group and three teeth in the PRP group did not have periapical radiolucid lesions before treatment, and one tooth in the BC group exhibited enlargement of a pre-existing periapical lesion after treatment, which was judged radiographically unsuccessful

^***^Three teeth in the PRP group and two in the BC group did not have periapical radiolucid lesions before treatment

^&^Total radiographic root area (RRA) was measured to comprehensively evaluate the development of tooth roots; root length and width could not be determined

^#^In this study, root development was measured using cone-beam CT (CBCT) images, but was not classified as excellent, good, satisfactory, or unsatisfactory

**Fig. 4:**
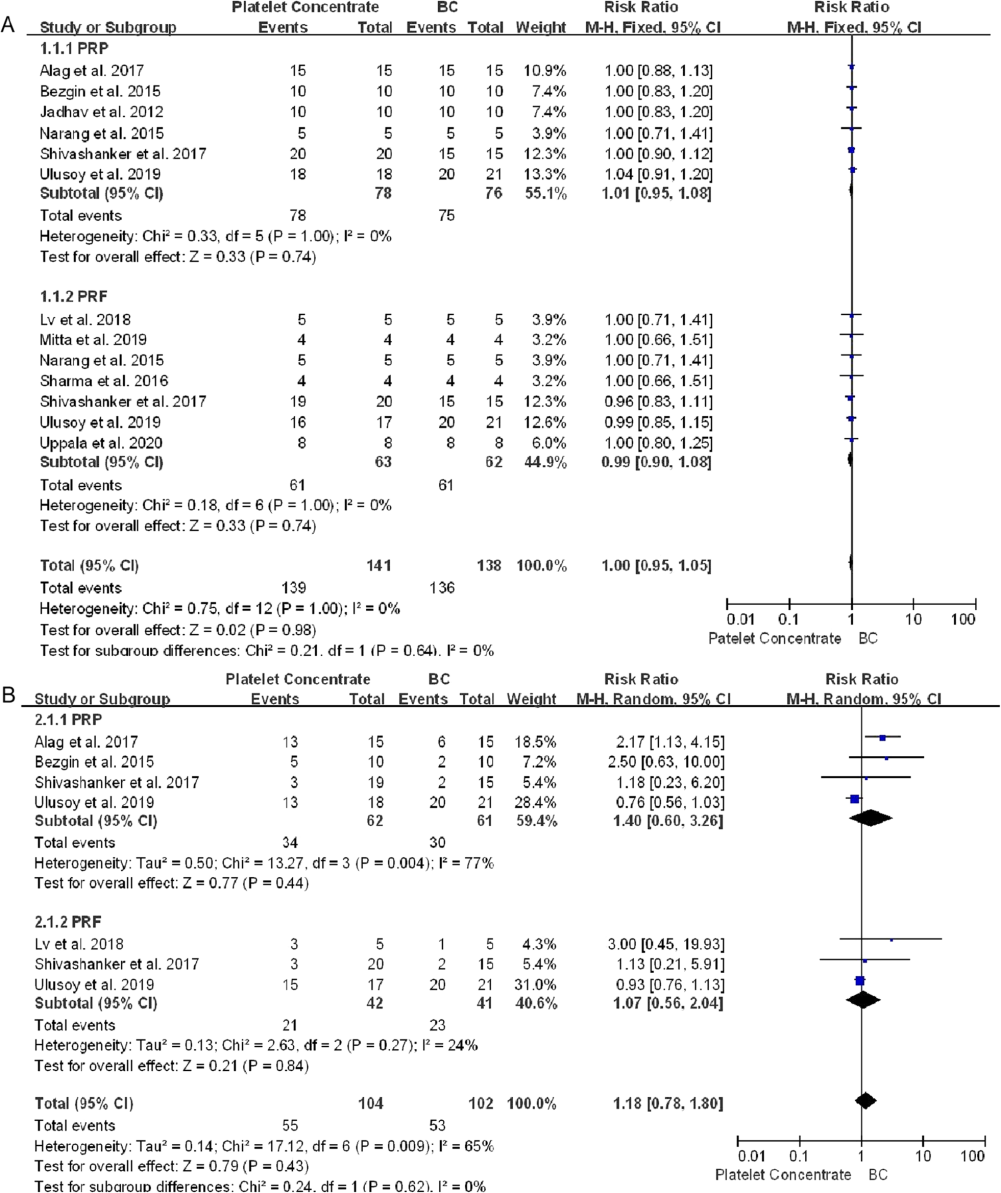

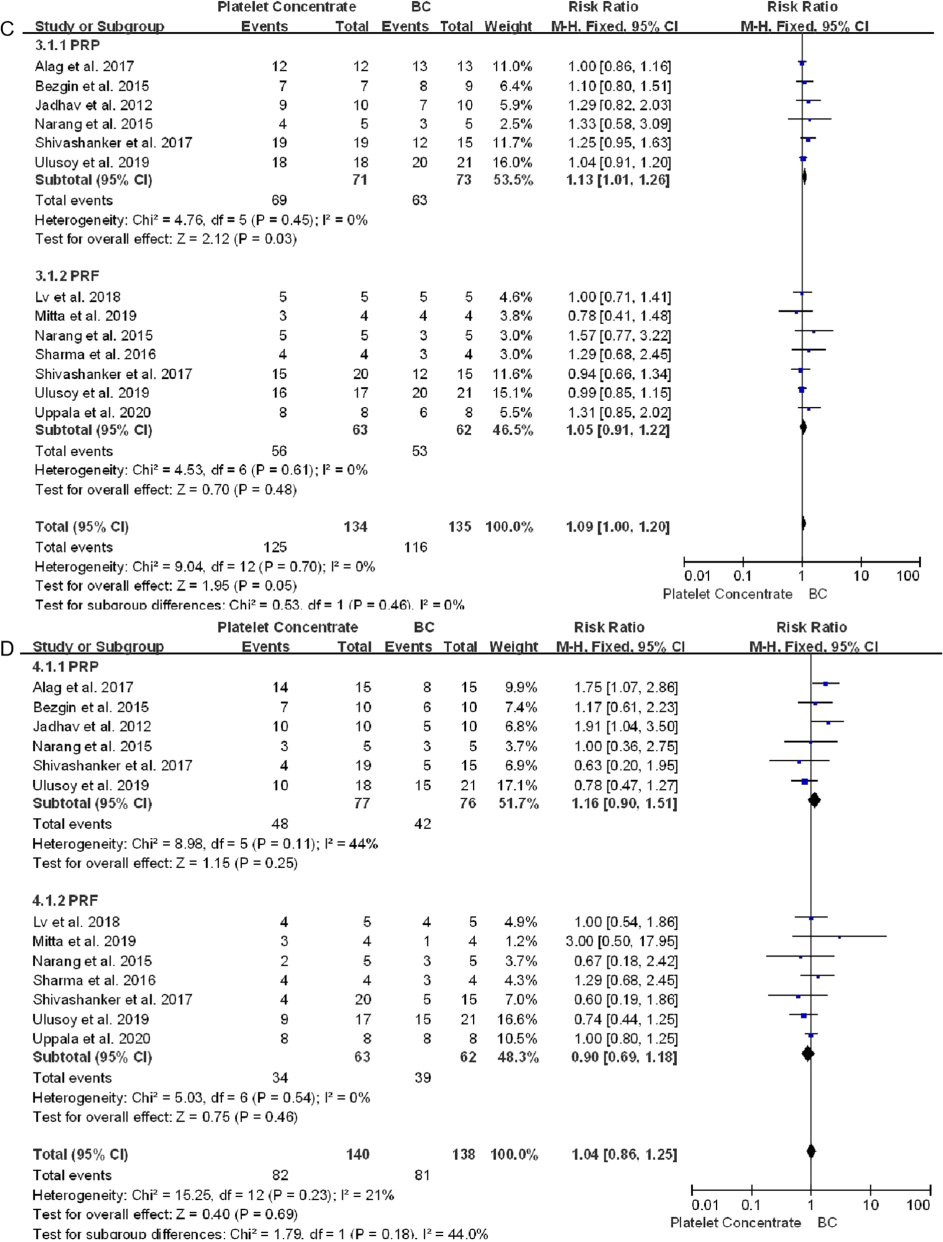

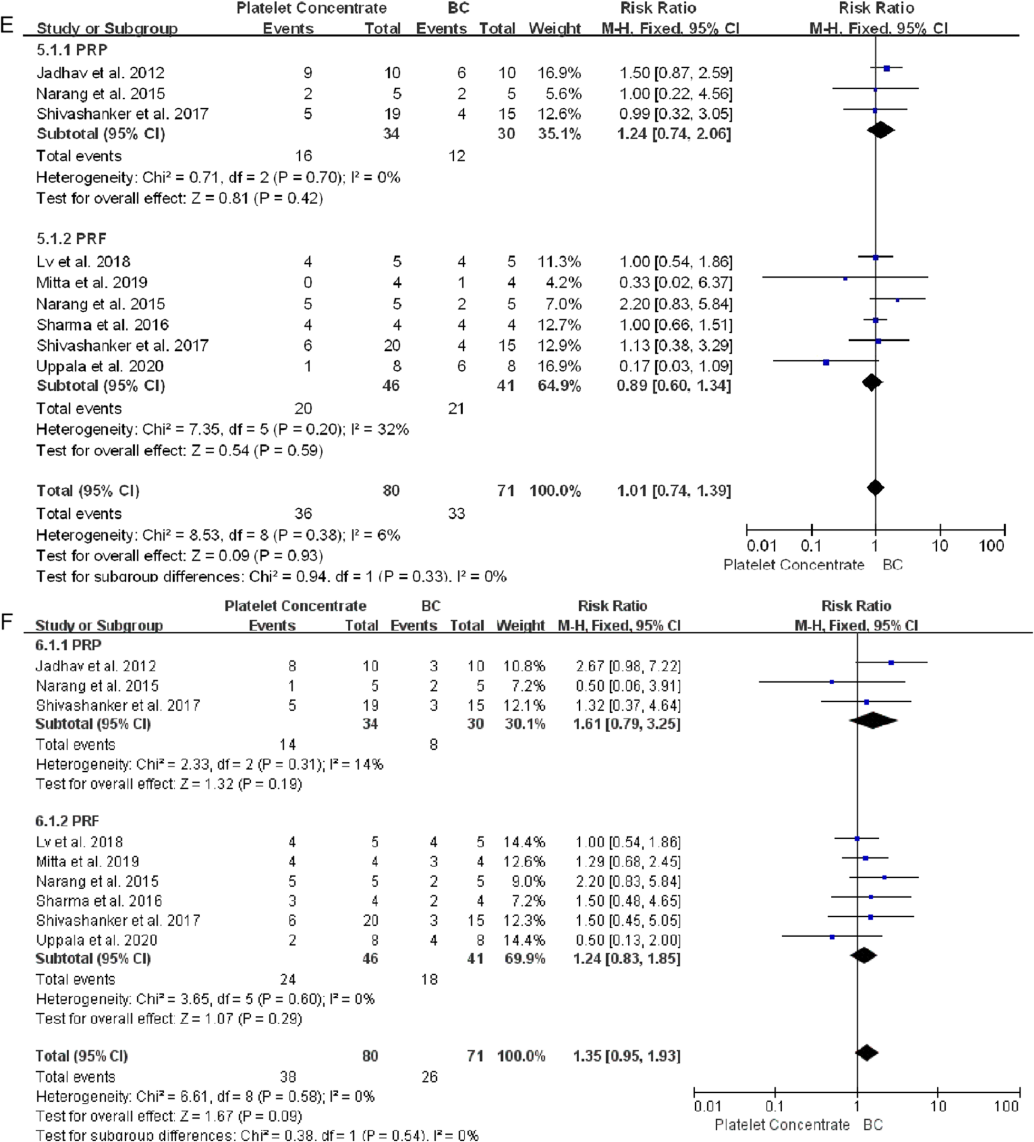
Forest plot showing factors associated with platelet concentrate and conventional blood clot scaffolds. **A** Clinical success. **B** Response to cold and electric pulp tests. **C** Periapical healing. **D** Apex closure. **E** Root lengthening. **F** Root canal thickening. No statistically significant between group differences were observed (*P* > 0.05 each) except for periapical healing in PRP vs BC groups (*P* = 0.03)

One article reported that one patient each in the PRF and BC groups showed signs and symptoms of failure, including spontaneous pain and extreme sensitivity to percussion, at 13 and 14 months, respectively [39]. Otherwise, all immature necrotic teeth treated with platelet concentrates and blood clots showed successful clinical outcomes during follow-up, with no significant differences between the platelet concentrate and BC groups (RR 1.00, 95% CI 0.95–1.05, *P* = 0.98) (Fig. 4A).

Five studies assessed responses to sensitivity pulp tests after treatment [14, 16, 32, 39, 41]. Fifty-five (52.9%) of the 104 teeth treated with platelet concentrates and 31 (47.9%) of the 66 teeth treated with BC showed positive responses to cold or electrical stimulation, with no significant difference between these groups (RR 1.18, 95% CI 0.78–1.80, *P* = 0.43) (Fig. 4B).

In all included articles, root development was assessed radiographically. Seven articles measured apical closure, thickness of the dentin walls and root lengthening [12, 15, 32–34, 38, 41], and two articles estimated the thickness of the dentin walls by measuring the percent increase in RRA [14, 39], with > 15% regarded as excellent, 5–15% as good, and < 5% as satisfactory [50]. One article [33] assessed periapical status using loose criteria [51], whereas another [16] measured root lengthening on CBCT images using the mean of raw data, but did not categorize findings as excellent, good, satisfactory and unsatisfactory.

Healing of periapical lesions did not differ significantly in teeth treated with platelet concentrates and blood clots (RR 1.09, 95% CI 1.00–1.20, *P* = 0.05) (Fig. 4C). In PRP group, teeth treated with PRP achieved better apical healing than BC group (RR 1.13, 95% CI 1.01–1.26, *P* = 0.03). Radiolucent areas were observed in two teeth treated with PRF group, and enlargement of a preexisting periapical lesion after treatment was observed in one control tooth, although these patients were clinically asymptomatic [14, 33]. Teeth treated with platelet concentrates achieved better apical closure (RR 1.04, 95% CI 0.86–1.25, *P* = 0.69), root lengthening (RR 1.01, 95% CI 0.74–1.39, *P* = 0.93) and thickening of the dentin walls (RR 1.35, 95% CI 0.95–1.93, *P* = 0.09) than teeth treated with BC, although these differences were not statistically significant (Fig. 4D–F).

### Synthesis of results (meta-analysis) under different influencing factors

In view of the patient's age, tooth type, etiology of pulp neurosis, mechanical instrumentation or not of the canal, root canal irrigation, and local anesthetics may have an impact on the therapeutic effect. They were analyzed separately. The meta-analysis results of patient age (< 18 years old), tooth type, and root canal invasive were similar to the subgroup analysis results based on the type of platelet concentrate used (Additional file 3, 4, 5). Other factors about etiology of pulp neurosis, root canal irrigation and local anesthetics, since fewer than three studies were included, meta-analysis could not be conducted (Additional file 6, 7).

### Sensitivity analysis and publication bias

**Fig. 5:**
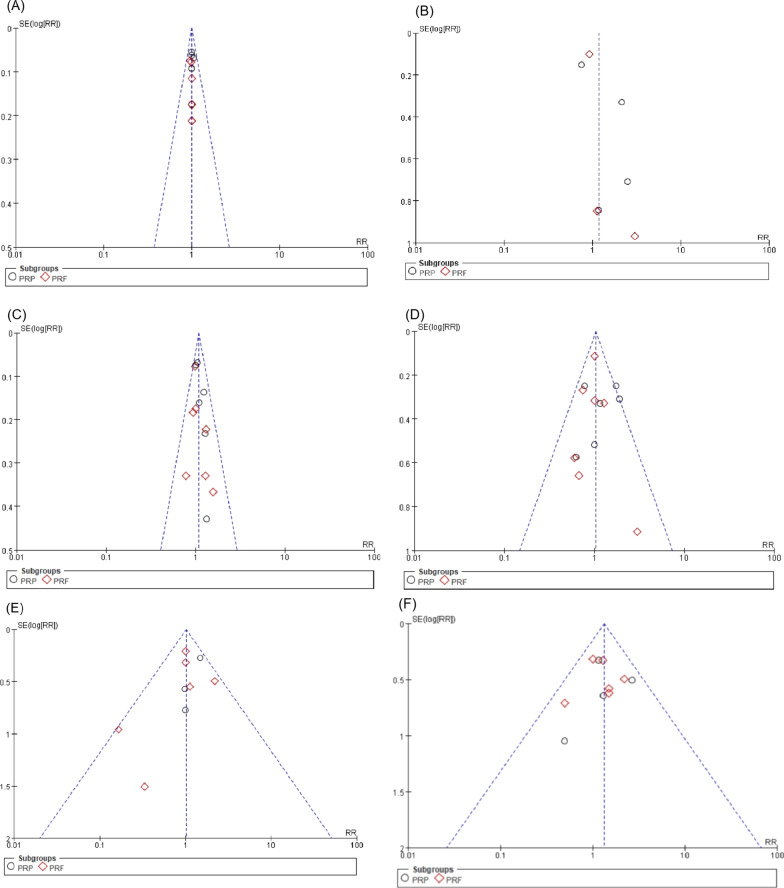
Funnel plot of comparison associated with platelet concentrate and conventional blood clot scaffolds. **A** Clinical success. **B** Response to cold and electric pulp tests. **C** Periapical healing. **D** Apex closure. **E** Root lengthening. **F** Root canal thickening. Funnel plot indicated that there were no obvious heterogeneity among the included studies

Sensitivity analysis showed that the combined effect amount did not change after excluding any study, suggesting that the results were reliable, except for the meta analysis of responses to sensitivity pulp tests. Funnel plots and Begg tests and Egg tests showed that there were no publication bias in the included literature (Fig. 5, Additional file 8, 9).

### Side effects of treatment

The main side effects reported in these articles were root canal calcification and tooth discoloration. One article reported partial pulp canal obliteration in four of ten teeth in both the treatment and control groups [14]. Discoloration after revascularization was observed in three articles [36, 40, 43], with one study [40] reporting a significantly higher incidence of crown discoloration in the BC group than in the PR group. No other adverse events were reported in immature necrotic teeth treated with PRP, PRF, or blood clots.

## Discussion

The present systematic review and meta-analysis identified few studies comparing platelet concentrations with blood clots in RE. Because systematic reviews are based on rigorous inclusion, exclusion and methodological criteria, few articles are available, with most studies to date being case reports or studies lacking a blood clot control group. Although these studies were not included in this review, all have reported that platelet concentrates have beneficial effects in the treatment of immature necrotic teeth [13, 18, 52–57]. Similar findings were observed in the present study, as it excluded articles that used alternative root canal scaffolds, such as Bio-Gide collagen membrane [58] or injectable scaffolds impregnated with basic fibroblast growth factor [59]. Additionally, the introduction of platelet concentrates in RE is relatively new, which may be another reason for the relatively limited research in this area.

The RoB varied among the included studies. In RCT studies, only one [33] was regarded as having a low RoB, whereas eight had an unclear [12, 14, 15, 32, 35, 38, 39, 41] and seven had a high [16, 34, 36, 37, 40, 42, 43] RoB. Because of the nature of the treatment, allocation could not be concealed and patients could not be blinded to treatment, as blood was drawn from the patients who received platelet concentrates. Owing to differences in operative protocols, clinicians could not be blinded to treatment in most of these studies, suggesting that a lack of blinding and concealment of allocation,which may not represent be serious sources of bias for the study outcomes. However, the small number of included studies may reduce the validity of the outcomes.

Of the 465 teeth included in the control and experimental groups of the analyzed studies, 457 (98.2%) remained clinically asymptomatic for the entire study duration. Only eight (1.8%) teeth showed signs and symptoms of failure, including spontaneous pain, sensitivity to percussion or reinfection [37, 38, 42]. This meta-analysis included eight RCT parallel studies [12, 14, 15, 32, 33, 35, 39, 42], one split-mouth study [16], and one case-control study [34]. Fifty-five (52.9%) of 104 teeth treated with platelet concentrates and 31 (47.0%) of 66 treated with blot clots displayed a positive response to cold or electrical stimulation (RR 1.18, 95% CI 0.78–1.80, *P* = 0.43). This response may be a clinical indicator of regenerative tissue status in root canals, but no direct correlation between pulp vitality and better root development has been reported to date. In addition, histological changes in the root canal were not analyzed in all of the included studies, precluding the determination of the nature of tissues formed in response to various revascularization techniques. Studies of whole dental pulp regeneration are needed. In addition, the sensitivity analysis found that the results would change significantly after excluding Ulusoy (Ulusoy et al.)[39] (Additional file 9). In this study, more than 80% of teeth showed a response to thermal and electric pulp tests, which was higer than other included studies, and it may be the main cause of heterogeneity. Due to the limited number of included literatures, they cannot be reevaluated by excluding literatures. The research results need more new epidemiological evidence for further verification.

Healing of the periapical lesion did not differ significantly in teeth treated with platelet concentrates and blood clots (RR 1.09, 95% CI 1.00–1.20, *P* = 0.05). However, in PRP group, teeth treated with PRP achieved better apical healing than BC group (RR 1.13, 95% CI 1.01–1.26, *P* = 0.03). In addition, healing times were shorter in PRP-treated teeth than in BC-treated teeth [14, 16], and periapical healing sizes after 6 months were smaller in PRP-treated teeth than in either PRF- or BC-treated teeth [14, 16], suggesting that PRP provides better and more rapid periapical wound healing than PRF or BC [33], PRP has the consistency of a liquid, enabling it to reach the periapex without any impedance [60]. By contrast, PRF has a gel-like consistency, delivering the maximum amount of growth factors to hasten the wound healing process. Moreover, teeth treated with platelet concentrates achieved better apex closure (RR 1.04, 95% CI 0.86–1.25, *P* = 0.69), root lengthening (RR 1.01, 95% CI 0.74–1.39, *P* = 0.93) and thickening of the dentin walls (RR 1.32, 95% CI 0.96–1.81,* P* = 0.08) than teeth treated with blood clots, although these differences were not statistically significant.

The present study had several limitations. First, radiographic methods differed among studies, directly affecting measurements of tooth lengthening and thickness of the dentin walls. Second, follow-up time differed among the included studies, and 12 or 18 months may not be sufficient to observe root maturogenesis, especially in teeth categorized as satisfactory.

## Conclusion

Platelet concentrate scaffold (PRP or PRF) assisted and traditional blood clot scaffold revascularization of non-vital immature permanent teeth diagnosed with necrotic pulpitis, resulted in similar outcomes. PRP provides better periapical healing than BC scaffold. In cases with periapical inflammation, using PRP as a scaffold may achieve better periapical healing. Platelet concentrate scaffolds can be used as an alternative for revascularization. Further studies with standardized protocols are necessary to assess the actual contribution of platelet concentrates to RE.

## Supplementary Information

**Additional file 1.** List of excluded trials with reasons (n = 7).

**Additional file 2.** Interexaminer and Intraexaminer Kappa Values for artical identification and screening, data extraction and quality assessment.

**Additional file 3.** The meta-analysis of the group of juvenile (< 18 years old).

**Additional file 4.** The forest map of studies involving only incisors.

**Additional file 5.** The forest map of studies involving mechanical instrumention or not of the canal.

**Additional file 6.** The factors of etiology of pulp necrosis.

**Additional file 7.** The study was divided into two groups for meta analysis according to whether EDTA was used.

**Additional file 8.** The Bgger's and Egg's test of included articals.

**Additional file 9.** The sensitivity analysis of included articals.

## Data Availability

The datasets used and/or analyzed during the current study are available from the corresponding author on reasonable request.

## Abbreviations

Ca(OH)_2_: Calcium hydroxide
MTA: Mineral trioxide aggregate
RE: Regenerative endodontics
3D: Three-dimensional
PRP: Platelet-rich plasma
PRF: Platelet-rich fibrin
P-PRF: Pure PRF
L-PRF: Leukocyte- and platelet-rich fibrin
A-PRF: Advanced PRF
i-PRF: Injectable PRF
RRs: Pooled Risk ratios
CGF: Concentrated growth factor
RoB: Risk of bias
CBCT: Cone-beam computed tomography
