# Supplementary material for: Are platelet concentrate scaffolds superior to traditional blood clot scaffolds in regeneration therapy of necrotic immature permanent teeth? A systematic review and meta-analysis
Source: BMC Oral Health. 2022 Dec 9;22:589. doi: 10.1186/s12903-022-02605-4 (PMC9733063; doi:10.1186/s12903-022-02605-4)
Supplement: Supplementary file 1 — Additional file 1. List of excluded trials with reasons (n = 7). [file 12903_2022_2605_MOESM1_ESM.pdf]

Additional file 1:

List of excluded trials with reasons (n=7)

Author	Title	reason for exclusion
Nageh et al. (2018)[17]	Assessment of Regaining Pulp Sensibility in Mature Necrotic Teeth Using a Modified Revascularization Technique with Platelet-rich Fibrin: A Clinical Study	Teeth are not immature teeth
Lu et al. (2018)[43]	Clinical study on autologus platelet-rich fibrin aided revascularization of immature permanent teeth	Publised in Chinese, non English
Santhakumar et al. (2018)[45]	A clinicoradiographic comparison of the effects of platelet-rich fibrin gel and platelet-rich fibrin membrane as scaffolds in the apexification treatment of young permanent teeth	No BC clot control group
Rizk et al. (2020)[46]	Comparative evaluation of Platelet Rich Plasma (PRP) versus Platelet Rich Fibrin (PRF) scaffolds in regenerative endodontic treatment of immature necrotic permanent maxillary central incisors: A double blinded randomized controlled trial.	No BC clot control group
Jayadevan et al. (2021)[18]	A comparative evaluation of Advanced Platelet-Rich Fibrin (A-PRF) and Platelet-Rich Fibrin (PRF) as a Scaffold in Regenerative Endodontic Treatment of Traumatized Immature Non-vital permanent anterior teeth: A Prospective clinical study	No BC clot control group
Kritika et al. (2021)[47]	Prospective cohort study of regenerative potential of non vital immature permanent maxillary central incisors using platelet rich fibrin scaffold	No BC clot control group
Liang et al. (2021)[44]	Efficacy of i-PRF in regenerative endodontics therapy for mature permanent teeth with pulp necrosis: study protocol for a multicentre randomised controlled trial	Teeth are not immature teeth
