# Supplementary material for: Are platelet concentrate scaffolds superior to traditional blood clot scaffolds in regeneration therapy of necrotic immature permanent teeth? A systematic review and meta-analysis
Source: BMC Oral Health. 2022 Dec 9;22:589. doi: 10.1186/s12903-022-02605-4 (PMC9733063; doi:10.1186/s12903-022-02605-4)
Supplement: Supplementary file 2 — Additional file 2. Interexaminer and Intraexaminer Kappa Values for artical identification and screening, data extraction and quality assessment. [file 12903_2022_2605_MOESM2_ESM.pdf]

Additional file 2. Interexaminer and Intraexaminer Kappa Values for article identification and screening, data extraction and quality assessment.

	Kappa	*P* value
article identification and screening (S Lin and L Ma)	0.894	.0001
data extraction(QW Tang and H Jin)	0.897	.0001
quality assessment(QW Tang and H Jin)	1.000	.0001
