# Supplementary material for: Are platelet concentrate scaffolds superior to traditional blood clot scaffolds in regeneration therapy of necrotic immature permanent teeth? A systematic review and meta-analysis
Source: BMC Oral Health. 2022 Dec 9;22:589. doi: 10.1186/s12903-022-02605-4 (PMC9733063; doi:10.1186/s12903-022-02605-4)
Supplement: Supplementary file 3 — Additional file 3. The meta-analysis of the group of juvenile (< 18 years old). [file 12903_2022_2605_MOESM3_ESM.pdf]

Additional file 3. The meta-analysis of the group of juvenile(<18 years old)

| | Studies | Participants | Statistical Method | Effect Estimate |
|---|---|---|---|---|
| 1.1 Clinical success | 4 | 137 | Risk Ratio (M-H, Fixed, 95% CI) | 1.01 [0.94, 1.09] |
| 1.1.1 PRP | 3 | 89 | Risk Ratio (M-H, Fixed, 95% CI) | 1.02 [0.94, 1.11] |
| 1.1.2 PRF | 2 | 48 | Risk Ratio (M-H, Fixed, 95% CI) | 0.99 [0.86, 1.14] |
| 2.1 Response to cold and electric pulp Test | 4 | 137 | Risk Ratio (M-H, Random, 95% CI) | 1.22 [0.75, 1.96] |
| 2.1.1 PRP | 3 | 89 | Risk Ratio (M-H, Random, 95% CI) | 1.46 [0.54, 3.99] |
| 2.1.2 PRF | 2 | 48 | Risk Ratio (M-H, Random, 95% CI) | 1.32 [0.34, 5.13] |
| 3.1 Periapical healing | 5 | 144 | Risk Ratio (M-H, Fixed, 95% CI) | 1.05 [0.96, 1.14] |
| 3.1.1 PRP | 3 | 80 | Risk Ratio (M-H, Fixed, 95% CI) | 1.04 [0.94, 1.15] |
| 3.1.2 PRF | 3 | 64 | Risk Ratio (M-H, Fixed, 95% CI) | 1.06 [0.91, 1.23] |
| 4.1 Apex closure | 4 | 137 | Risk Ratio (M-H, Fixed, 95% CI) | 1.01 [0.79, 1.29] |
| 4.1.1 PRP | 3 | 89 | Risk Ratio (M-H, Fixed, 95% CI) | 1.14 [0.84, 1.54] |
| 4.1.2 PRF | 2 | 48 | Risk Ratio (M-H, Fixed, 95% CI) | 0.80 [0.53, 1.22] |
| 5.1 Root lengthening | 2 | 26 | Risk Ratio (M-H, Random, 95% CI) | 0.46 [0.04, 5.18] |
| 5.1.1 PRP | 0 | 0 | Risk Ratio (M-H, Random, 95% CI) | Not estimable |
| 5.1.2 PRF | 26 | 26 | Risk Ratio (M-H, Random, 95% CI) | 0.46 [0.04, 5.18] |
| 6.1 Root canal thickening | 3 | 46 | Risk Ratio (M-H, Fixed, 95% CI) | 0.93 [0.58, 1.48] |
| 6.1.1 PRP | 1 | 20 | Risk Ratio (M-H, Fixed, 95% CI) | 1.17 [0.61, 2.23] |
| 6.1.2 PRF | 2 | 26 | Risk Ratio (M-H, Fixed, 95% CI) | 0.75 [0.38, 1.46] |
