# Supplementary material for: Are platelet concentrate scaffolds superior to traditional blood clot scaffolds in regeneration therapy of necrotic immature permanent teeth? A systematic review and meta-analysis
Source: BMC Oral Health. 2022 Dec 9;22:589. doi: 10.1186/s12903-022-02605-4 (PMC9733063; doi:10.1186/s12903-022-02605-4)

Additional file 4. The forest map of studies involving only incisors.

(A) Clinical success

Study or Subgroup	Platelet Concentrate Events	Platelet Concentrate Total	BC Events	BC Total	Weight	Risk Ratio M-H, Fixed, 95% CI
**1.1.1 PRP**						
Jadhav et al. 2012	10	10	10	10	16.2%	1.00 [0.83, 1.20]
Ulusoy et al. 2019	18	18	20	21	29.3%	1.04 [0.91, 1.20]
**Subtotal (95% CI)**		**28**		**31**	**45.5%**	**1.03 [0.92, 1.15]**
Total events	28		30			
Heterogeneity: Chi² = 0.14, df = 1 (P = 0.70); I² = 0%						
Test for overall effect: Z = 0.52 (P = 0.61)						
**1.1.2 PRF**						
Mitta et al. 2019	4	4	4	4	6.9%	1.00 [0.66, 1.51]
Sharma et al. 2016	4	4	4	4	6.9%	1.00 [0.66, 1.51]
Ulusoy et al. 2019	16	17	20	21	27.6%	0.99 [0.85, 1.15]
Uppala et al. 2020	8	8	8	8	13.1%	1.00 [0.80, 1.25]
**Subtotal (95% CI)**		**33**		**37**	**54.5%**	**0.99 [0.88, 1.12]**
Total events	32		36			
Heterogeneity: Chi² = 0.01, df = 3 (P = 1.00); I² = 0%						
Test for overall effect: Z = 0.10 (P = 0.92)						
**Total (95% CI)**		**61**		**68**	**100.0%**	**1.01 [0.93, 1.10]**
Total events	60		66			
Heterogeneity: Chi² = 0.35, df = 5 (P = 1.00); I² = 0%						
Test for overall effect: Z = 0.24 (P = 0.81)						
Test for subgroup differences: Chi² = 0.17, df = 1 (P = 0.68), I² = 0%						

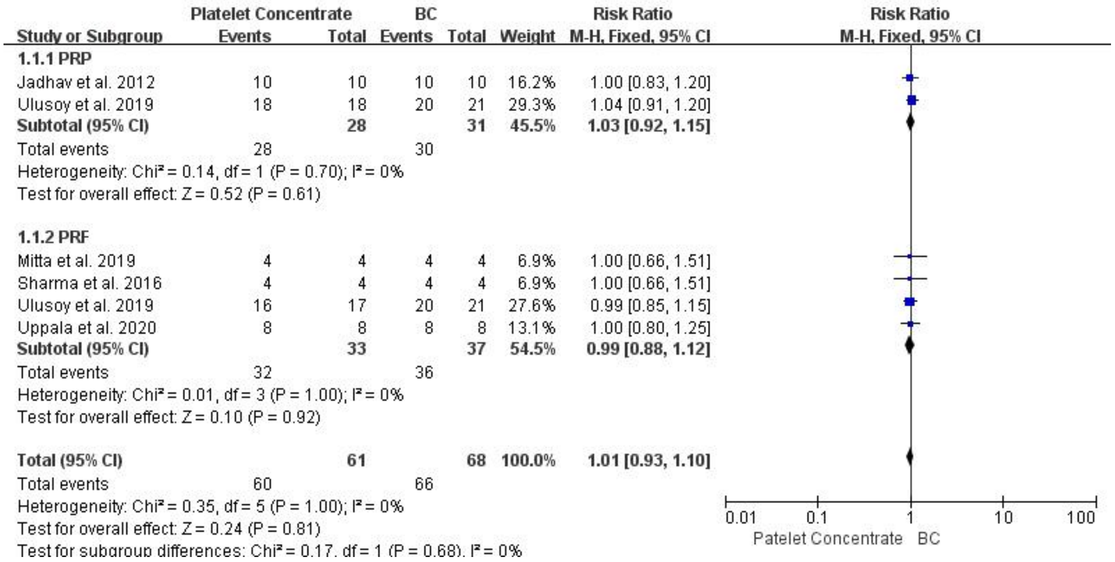

(B) Response to cold and electric pulp tests.

Study or Subgroup	Platelet Concentrate Events	Platelet Concentrate Total	BC Events	BC Total	Weight	Risk Ratio M-H, Random, 95% CI
**2.1.1 PRP**						
Ulusoy et al. 2019	13	18	20	21	35.4%	0.76 [0.56, 1.03]
**Subtotal (95% CI)**		**18**		**21**	**35.4%**	**0.76 [0.56, 1.03]**
Total events	13		20			
Heterogeneity: Not applicable						
Test for overall effect: Z = 1.80 (P = 0.07)						
**2.1.2 PRF**						
Ulusoy et al. 2019	15	17	20	21	64.6%	0.93 [0.76, 1.13]
**Subtotal (95% CI)**		**17**		**21**	**64.6%**	**0.93 [0.76, 1.13]**
Total events	15		20			
Heterogeneity: Not applicable						
Test for overall effect: Z = 0.76 (P = 0.45)						
**Total (95% CI)**		**35**		**42**	**100.0%**	**0.86 [0.71, 1.06]**
Total events	28		40			
Heterogeneity: Tau² = 0.01; Chi² = 1.37, df = 1 (P = 0.24); I² = 27%						
Test for overall effect: Z = 1.43 (P = 0.15)						
Test for subgroup differences: Chi² = 1.18, df = 1 (P = 0.28), I² = 15.3%						

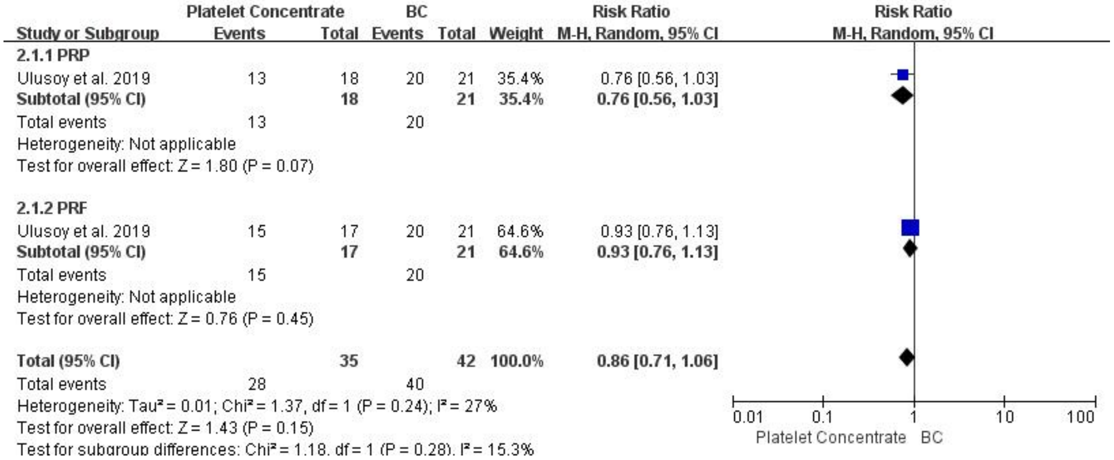

(C) Periapical healing

Study or Subgroup	Platelet Concentrate Events	Platelet Concentrate Total	BC Events	BC Total	Weight	Risk Ratio M-H, Fixed, 95% CI
**3.1.1 PRP**						
Jadhav et al. 2012	9	10	7	10	12.0%	1.29 [0.82, 2.03]
Ulusoy et al. 2019	18	18	20	21	32.5%	1.04 [0.91, 1.20]
**Subtotal (95% CI)**		**28**		**31**	**44.5%**	**1.11 [0.94, 1.31]**
Total events	27		27			
Heterogeneity: Chi² = 1.16, df = 1 (P = 0.28); I² = 14%						
Test for overall effect: Z = 1.25 (P = 0.21)						
**3.1.2 PRF**						
Mitta et al. 2019	3	4	4	4	7.7%	0.78 [0.41, 1.48]
Sharma et al. 2016	4	4	3	4	6.0%	1.29 [0.68, 2.45]
Ulusoy et al. 2019	16	17	20	21	30.6%	0.99 [0.85, 1.15]
Uppala et al. 2020	8	8	6	8	11.1%	1.31 [0.85, 2.02]
**Subtotal (95% CI)**		**33**		**37**	**55.5%**	**1.06 [0.89, 1.25]**
Total events	31		33			
Heterogeneity: Chi² = 2.87, df = 3 (P = 0.41); I² = 0%						
Test for overall effect: Z = 0.63 (P = 0.53)						
**Total (95% CI)**		**61**		**68**	**100.0%**	**1.08 [0.96, 1.21]**
Total events	58		60			
Heterogeneity: Chi² = 4.10, df = 5 (P = 0.53); I² = 0%						
Test for overall effect: Z = 1.28 (P = 0.20)						
Test for subgroup differences: Chi² = 0.18, df = 1 (P = 0.67), I² = 0%						

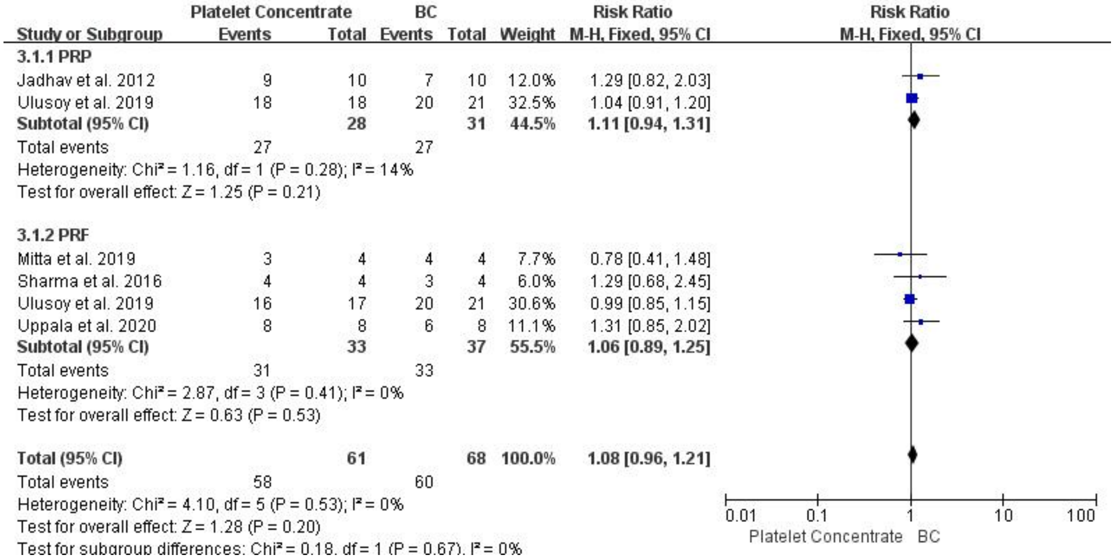

(D) Apex closure

Study or Subgroup	Platelet Concentrate Events	Platelet Concentrate Total	BC Events	BC Total	Weight	Risk Ratio M-H, Fixed, 95% CI
**4.1.1 PRP**						
Jadhav et al. 2012	10	10	5	10	12.0%	1.91 [1.04, 3.50]
Ulusoy et al. 2019	10	18	15	21	30.3%	0.78 [0.47, 1.27]
**Subtotal (95% CI)**		**28**		**31**	**42.3%**	**1.10 [0.76, 1.60]**
Total events	20		20			
Heterogeneity: Chi² = 5.08, df = 1 (P = 0.02); I² = 80%						
Test for overall effect: Z = 0.50 (P = 0.62)						
**4.1.2 PRF**						
Mitta et al. 2019	3	4	1	4	2.2%	3.00 [0.50, 17.95]
Sharma et al. 2016	4	4	3	4	7.6%	1.29 [0.68, 2.45]
Ulusoy et al. 2019	9	17	15	21	29.3%	0.74 [0.44, 1.25]
Uppala et al. 2020	8	8	8	8	18.6%	1.00 [0.80, 1.25]
**Subtotal (95% CI)**		**33**		**37**	**57.7%**	**0.98 [0.74, 1.31]**
Total events	24		27			
Heterogeneity: Chi² = 3.30, df = 3 (P = 0.35); I² = 9%						
Test for overall effect: Z = 0.12 (P = 0.90)						
**Total (95% CI)**		**61**		**68**	**100.0%**	**1.03 [0.82, 1.30]**
Total events	44		47			
Heterogeneity: Chi² = 8.66, df = 5 (P = 0.12); I² = 42%						
Test for overall effect: Z = 0.27 (P = 0.79)						
Test for subgroup differences: Chi² = 0.22, df = 1 (P = 0.64), I² = 0%						

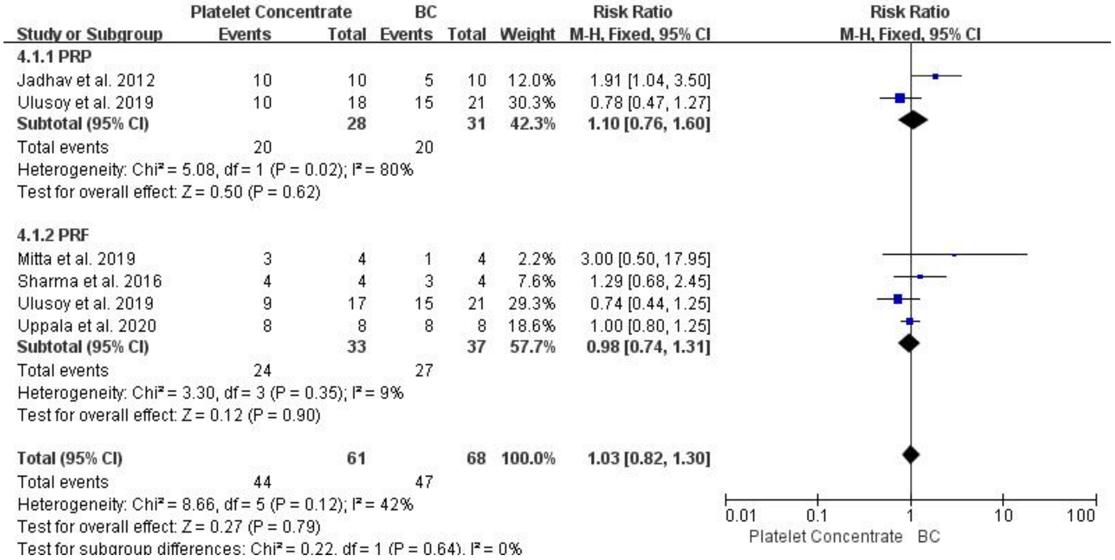

(E) Root lengthening

Study or Subgroup	Platelet Concentrate Events	Platelet Concentrate Total	BC Events	BC Total	Weight	Risk Ratio M-H, Random, 95% CI
**5.1.1 PRP**						
Jadhav et al. 2012	9	10	6	10	39.3%	1.50 [0.87, 2.59]
**Subtotal (95% CI)**		**10**		**10**	**39.3%**	**1.50 [0.87, 2.59]**
Total events	9		6			
Heterogeneity: Not applicable						
Test for overall effect: Z = 1.45 (P = 0.15)						
**5.1.2 PRF**						
Mitta et al. 2019	0	4	1	4	5.6%	0.33 [0.02, 6.37]
Sharma et al. 2016	4	4	4	4	43.2%	1.00 [0.66, 1.51]
Uppala et al. 2020	1	8	6	8	11.9%	0.17 [0.03, 1.09]
**Subtotal (95% CI)**		**16**		**16**	**60.7%**	**0.42 [0.04, 4.49]**
Total events	5		11			
Heterogeneity: Tau² = 3.51; Chi² = 12.20, df = 2 (P = 0.002); I² = 84%						
Test for overall effect: Z = 0.72 (P = 0.47)						
**Total (95% CI)**		**26**		**26**	**100.0%**	**0.89 [0.42, 1.87]**
Total events	14		17			
Heterogeneity: Tau² = 0.29; Chi² = 8.39, df = 3 (P = 0.04); I² = 64%						
Test for overall effect: Z = 0.31 (P = 0.76)						
Test for subgroup differences: Chi² = 1.06, df = 1 (P = 0.30), I² = 5.6%						

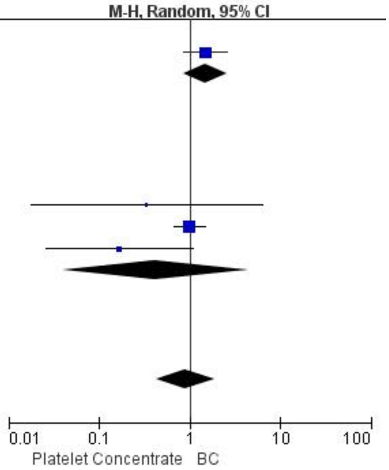

(F) Root canal thickening

Study or Subgroup	Platelet Concentrate Events	Platelet Concentrate Total	BC Events	BC Total	Weight	Risk Ratio M-H, Fixed, 95% CI
**6.1.1 PRP**						
Jadhav et al. 2012	8	10	3	10	24.0%	2.67 [0.98, 7.22]
**Subtotal (95% CI)**		**10**		**10**	**24.0%**	**2.67 [0.98, 7.22]**
Total events	8		3			
Heterogeneity: Not applicable						
Test for overall effect: Z = 1.93 (P = 0.05)						
**6.1.2 PRF**						
Mitta et al. 2019	4	4	3	4	28.0%	1.29 [0.68, 2.45]
Sharma et al. 2016	3	4	2	4	16.0%	1.50 [0.48, 4.65]
Uppala et al. 2020	2	8	4	8	32.0%	0.50 [0.13, 2.00]
**Subtotal (95% CI)**		**16**		**16**	**76.0%**	**1.00 [0.56, 1.77]**
Total events	9		9			
Heterogeneity: Chi² = 2.04, df = 2 (P = 0.36); I² = 2%						
Test for overall effect: Z = 0.00 (P = 1.00)						
**Total (95% CI)**		**26**		**26**	**100.0%**	**1.40 [0.85, 2.29]**
Total events	17		12			
Heterogeneity: Chi² = 3.81, df = 3 (P = 0.28); I² = 21%						
Test for overall effect: Z = 1.33 (P = 0.18)						
Test for subgroup differences: Chi² = 2.80, df = 1 (P = 0.09), I² = 64.3%						

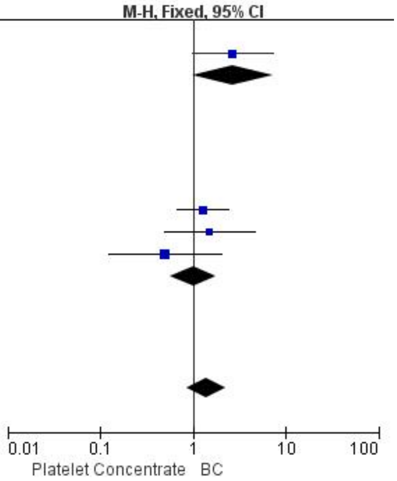

Supplement: Supplementary file 4 — Additional file 4. The forest map of studies involving only incisors. [file 12903_2022_2605_MOESM4_ESM.pdf]
