# Supplementary material for: Are platelet concentrate scaffolds superior to traditional blood clot scaffolds in regeneration therapy of necrotic immature permanent teeth? A systematic review and meta-analysis
Source: BMC Oral Health. 2022 Dec 9;22:589. doi: 10.1186/s12903-022-02605-4 (PMC9733063; doi:10.1186/s12903-022-02605-4)
Supplement: Supplementary file 5 — Additional file 5. The forest map of studies involving mechanical instrumention or not of the canal. [file 12903_2022_2605_MOESM5_ESM.pdf]

Additional file 5. The forest map of studies involving imechanical instrumentation or not of the canal.

（A）Clinical success

Minimal instrumentation

Study or Subgroup	Platelet Concentrate Events	Platelet Concentrate Total	BC Events	BC Total	Weight	Odds Ratio M-H, Random, 95% CI
**1.2.1 PRR**						
Jadhav et al. 2012	10	10	10	10		Not estimable
Narang et al. 2015	5	5	5	5		Not estimable
**Subtotal (95% CI)**		**15**		**15**		**Not estimable**
Total events	15		15			
Heterogeneity: Not applicable						
Test for overall effect: Not applicable						
**1.2.2 PRF**						
Mitta et al. 2019	4	4	4	4		Not estimable
Narang et al. 2015	5	5	5	5		Not estimable
Sharma et al. 2016	4	4	4	4		Not estimable
Shivashanker et al. 2017	19	20	15	15	100.0%	0.42 [0.02, 11.03]
**Subtotal (95% CI)**		**33**		**28**	**100.0%**	**0.42 [0.02, 11.03]**
Total events	32		28			
Heterogeneity: Not applicable						
Test for overall effect: Z = 0.52 (P = 0.60)						
**Total (95% CI)**		**48**		**43**	**100.0%**	**0.42 [0.02, 11.03]**
Total events	47		43			
Heterogeneity: Not applicable						
Test for overall effect: Z = 0.52 (P = 0.60)						
Test for subgroup differences: Not applicable						

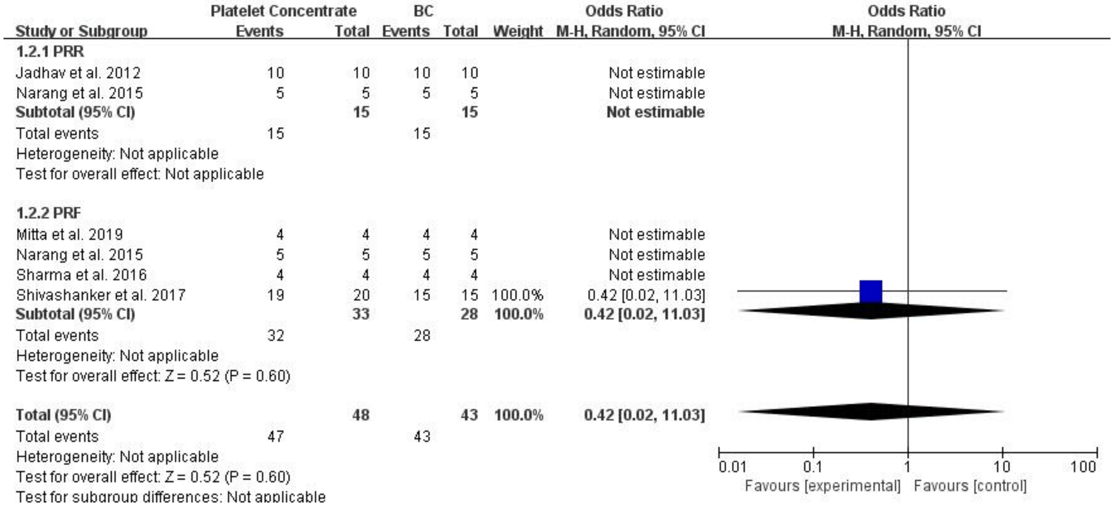

No instrumentation

Study or Subgroup	Platelet Concentrate Events	Platelet Concentrate Total	BC Events	BC Total	Weight	Odds Ratio M-H, Fixed, 95% CI
**1.3.1 PRR**						
Alag et al. 2017	15	15	15	15		Not estimable
Bezgin et al. 2015	10	10	10	10		Not estimable
Ulusoy et al. 2019	18	18	20	21	32.2%	2.71 [0.10, 70.65]
**Subtotal (95% CI)**		**43**		**46**	**32.2%**	**2.71 [0.10, 70.65]**
Total events	43		45			
Heterogeneity: Not applicable						
Test for overall effect: Z = 0.60 (P = 0.55)						
**1.3.2 PRF**						
Lv et al. 2018	5	5	5	5		Not estimable
Ulusoy et al. 2019	16	17	20	21	67.8%	0.80 [0.05, 13.81]
**Subtotal (95% CI)**		**22**		**26**	**67.8%**	**0.80 [0.05, 13.81]**
Total events	21		25			
Heterogeneity: Not applicable						
Test for overall effect: Z = 0.15 (P = 0.88)						
**Total (95% CI)**		**65**		**72**	**100.0%**	**1.41 [0.18, 11.17]**
Total events	64		70			
Heterogeneity: Chi² = 0.31, df = 1 (P = 0.58); I² = 0%						
Test for overall effect: Z = 0.33 (P = 0.74)						
Test for subgroup differences: Chi² = 0.30, df = 1 (P = 0.58), I² = 0%						

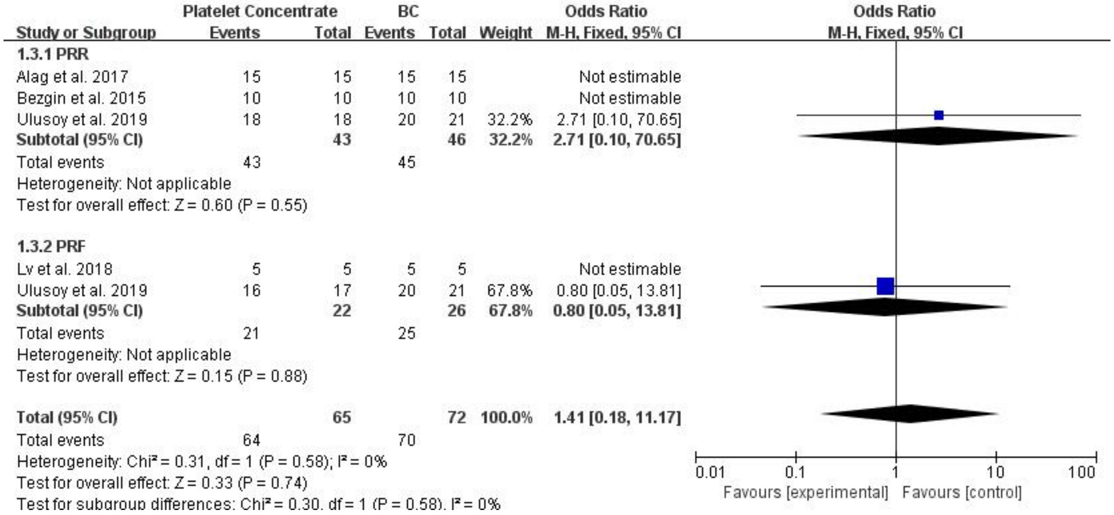

(B) Response to cold and electric pulp tests.

No instrumentation

Study or Subgroup	Platelet Concentrate Events	Platelet Concentrate Total	BC Events	BC Total	Weight	Odds Ratio M-H, Fixed, 95% CI
**2.3.1 PRR**						
Alag et al. 2017	13	15	6	15	8.5%	9.75 [1.59, 59.70]
Bezgin et al. 2015	5	10	2	10	10.6%	4.00 [0.55, 29.10]
Ulusoy et al. 2019	13	18	20	21	54.4%	0.13 [0.01, 1.24]
**Subtotal (95% CI)**		**43**		**46**	**73.4%**	**1.80 [0.72, 4.48]**
Total events	31		28			
Heterogeneity: Chi² = 9.17, df = 2 (P = 0.01); I² = 78%						
Test for overall effect: Z = 1.26 (P = 0.21)						
**2.3.2 PRF**						
Lv et al. 2018	3	5	1	5	4.2%	6.00 [0.35, 101.57]
Ulusoy et al. 2019	15	17	20	21	22.3%	0.38 [0.03, 4.53]
**Subtotal (95% CI)**		**22**		**26**	**26.6%**	**1.27 [0.25, 6.60]**
Total events	18		21			
Heterogeneity: Chi² = 2.08, df = 1 (P = 0.15); I² = 52%						
Test for overall effect: Z = 0.29 (P = 0.77)						
**Total (95% CI)**		**65**		**72**	**100.0%**	**1.66 [0.75, 3.68]**
Total events	49		49			
Heterogeneity: Chi² = 11.47, df = 4 (P = 0.02); I² = 65%						
Test for overall effect: Z = 1.25 (P = 0.21)						
Test for subgroup differences: Chi² = 0.13, df = 1 (P = 0.72), I² = 0%						

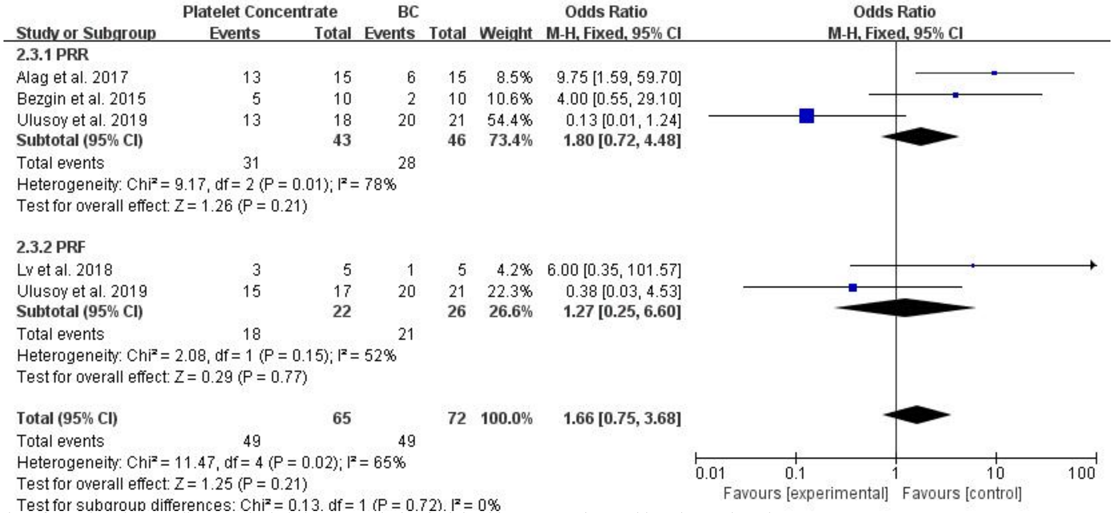

Since fewer than three studies were included, meta-analysis of minimal instrumentation could not be conducted.

(C) Periapical healing.

Minimal instrumentation

Study or Subgroup	Platelet Concentrate Events	Platelet Concentrate Total	BC Events	BC Total	Weight	Odds Ratio M-H, Fixed, 95% CI
**3.2.2 PRR**						
Jadhav et al. 2012	9	10	7	10	9.4%	3.86 [0.33, 45.57]
Narang et al. 2015	4	5	3	5	8.1%	2.67 [0.16, 45.14]
Shivashanker et al. 2017	19	19	12	15	4.7%	10.92 [0.52, 229.91]
**Subtotal (95% CI)**		**34**		**30**	**22.2%**	**4.91 [1.05, 23.02]**
Total events	32		22			
Heterogeneity: Chi² = 0.48, df = 2 (P = 0.79); I² = 0%						
Test for overall effect: Z = 2.02 (P = 0.04)						
**3.2.3 PRF**						
Mitta et al. 2019	3	4	4	4	18.2%	0.26 [0.01, 8.52]
Narang et al. 2015	5	5	3	5	3.9%	7.86 [0.28, 217.11]
Sharma et al. 2016	4	4	3	4	4.7%	3.86 [0.12, 126.73]
Shivashanker et al. 2017	15	20	12	15	46.2%	0.75 [0.15, 3.79]
Uppala et al. 2020	8	8	6	8	4.9%	6.54 [0.27, 160.97]
**Subtotal (95% CI)**		**41**		**36**	**77.8%**	**1.54 [0.55, 4.35]**
Total events	35		28			
Heterogeneity: Chi² = 3.73, df = 4 (P = 0.44); I² = 0%						
Test for overall effect: Z = 0.82 (P = 0.41)						
**Total (95% CI)**		**75**		**66**	**100.0%**	**2.29 [0.99, 5.30]**
Total events	67		50			
Heterogeneity: Chi² = 5.54, df = 7 (P = 0.59); I² = 0%						
Test for overall effect: Z = 1.94 (P = 0.05)						
Test for subgroup differences: Chi² = 1.49, df = 1 (P = 0.22), I² = 32.8%						

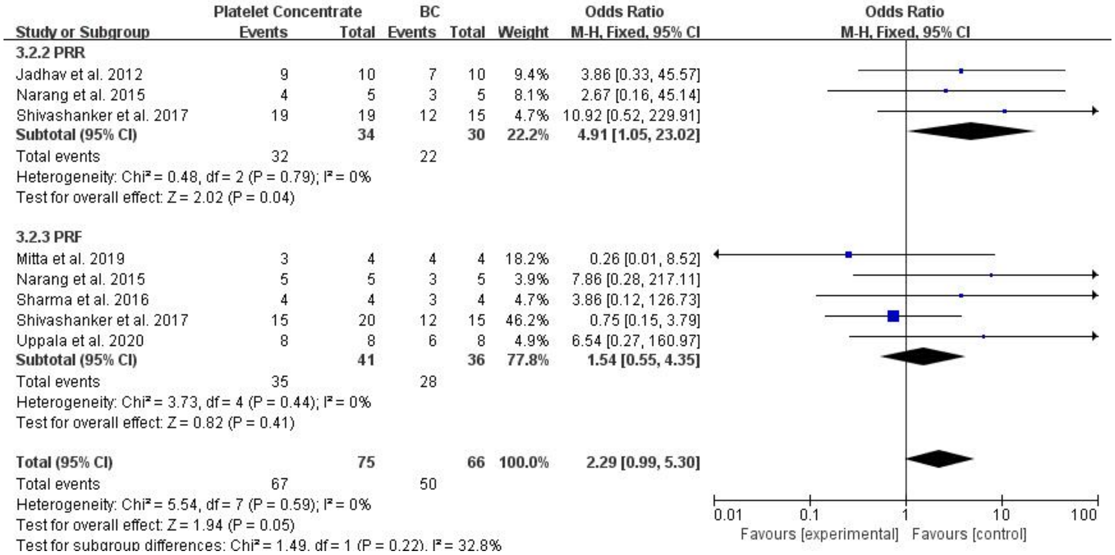

No instrumentation

Study or Subgroup	Platelet Concentrate Events	Platelet Concentrate Total	BC Events	BC Total	Weight	Odds Ratio M-H, Fixed, 95% CI
**3.3.1 PRR**						
Alag et al. 2017	12	12	13	13		Not estimable
Bezgin et al. 2015	7	7	8	9	31.0%	2.65 [0.09, 75.29]
**Subtotal (95% CI)**		**19**		**22**	**31.0%**	**2.65 [0.09, 75.29]**
Total events	19		21			
Heterogeneity: Not applicable						
Test for overall effect: Z = 0.57 (P = 0.57)						
**3.3.2 PRF**						
Lv et al. 2018	5	5	5	5		Not estimable
Ulusoy et al. 2019	16	17	20	21	69.0%	0.80 [0.05, 13.81]
**Subtotal (95% CI)**		**22**		**26**	**69.0%**	**0.80 [0.05, 13.81]**
Total events	21		25			
Heterogeneity: Not applicable						
Test for overall effect: Z = 0.15 (P = 0.88)						
**Total (95% CI)**		**41**		**48**	**100.0%**	**1.37 [0.17, 11.16]**
Total events	40		46			
Heterogeneity: Chi² = 0.29, df = 1 (P = 0.59); I² = 0%						
Test for overall effect: Z = 0.30 (P = 0.77)						
Test for subgroup differences: Chi² = 0.28, df = 1 (P = 0.59), I² = 0%						

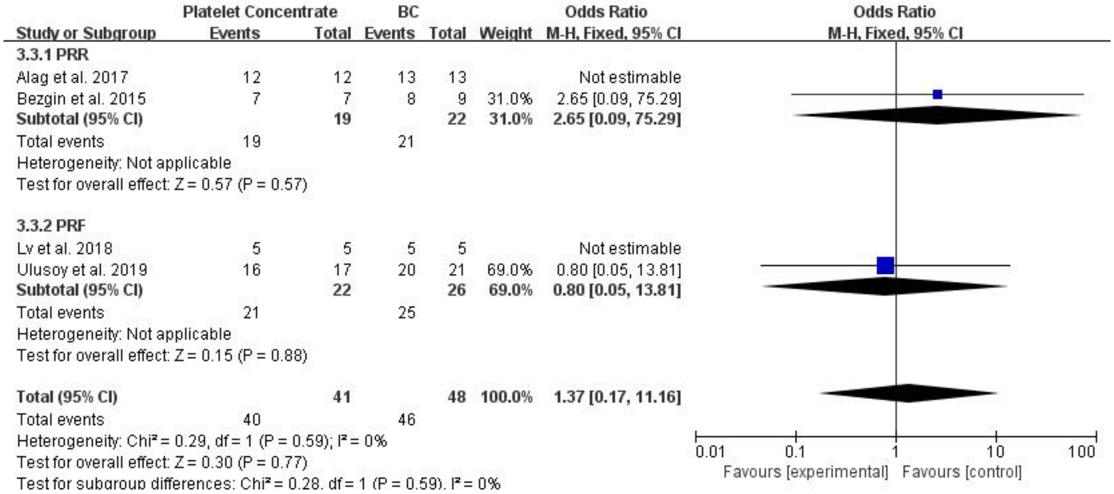

(D) Apex closure.

Minimal instrumentation

Study or Subgroup	Platelet Concentrate Events	Platelet Concentrate Total	BC Events	BC Total	Weight	Odds Ratio M-H, Random, 95% CI
**4.2.1 PRP**						
Jadhav et al. 2012	10	10	5	10	9.3%	21.00 [0.97, 453.91]
Narang et al. 2015	3	5	3	5	12.7%	1.00 [0.08, 12.56]
Shivashanker et al. 2017	4	19	5	15	24.5%	0.53 [0.11, 2.49]
**Subtotal (95% CI)**		**34**		**30**	**46.5%**	**1.64 [0.21, 12.67]**
Total events	17		13			
Heterogeneity: Tau² = 1.87; Chi² = 4.67, df = 2 (P = 0.10); I² = 57%						
Test for overall effect: Z = 0.47 (P = 0.64)						
**4.2.2 PRF**						
Mitta et al. 2019	3	4	1	4	8.7%	9.00 [0.37, 220.93]
Narang et al. 2015	2	5	3	5	12.7%	0.44 [0.04, 5.58]
Sharma et al. 2016	4	4	3	4	7.5%	3.86 [0.12, 126.73]
Shivashanker et al. 2017	4	20	5	15	24.6%	0.50 [0.11, 2.32]
Uppala et al. 2020	8	8	8	8		Not estimable
**Subtotal (95% CI)**		**41**		**36**	**53.5%**	**0.98 [0.26, 3.64]**
Total events	21		20			
Heterogeneity: Tau² = 0.30; Chi² = 3.54, df = 3 (P = 0.32); I² = 15%						
Test for overall effect: Z = 0.03 (P = 0.98)						
**Total (95% CI)**		**75**		**66**	**100.0%**	**1.16 [0.41, 3.24]**
Total events	38		33			
Heterogeneity: Tau² = 0.51; Chi² = 8.23, df = 6 (P = 0.22); I² = 27%						
Test for overall effect: Z = 0.28 (P = 0.78)						
Test for subgroup differences: Chi² = 0.17, df = 1 (P = 0.68), I² = 0%						

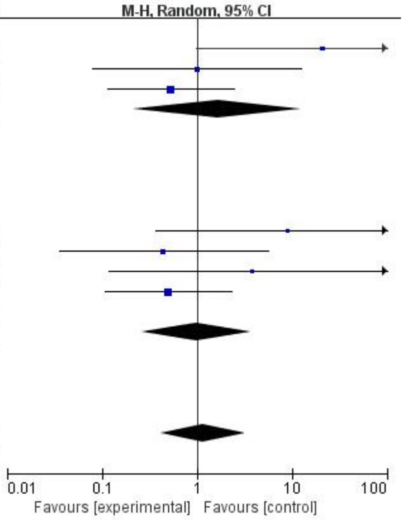

No instrumentation

Study or Subgroup	Platelet Concentrate Events	Platelet Concentrate Total	BC Events	BC Total	Weight	Odds Ratio M-H, Random, 95% CI
**4.3.1 PRP**						
Alag et al. 2017	14	15	8	15	28.1%	12.25 [1.27, 118.36]
Bezgin et al. 2015	7	10	6	10	32.8%	1.56 [0.24, 9.91]
**Subtotal (95% CI)**		**25**		**25**	**61.0%**	**3.92 [0.52, 29.74]**
Total events	21		14			
Heterogeneity: Tau² = 1.04; Chi² = 1.93, df = 1 (P = 0.16); I² = 48%						
Test for overall effect: Z = 1.32 (P = 0.19)						
**4.3.2 PRF**						
Lv et al. 2018	4	4	5	4		Not estimable
Ulusoy et al. 2019	9	17	15	21	39.0%	0.45 [0.12, 1.72]
**Subtotal (95% CI)**		**21**		**25**	**39.0%**	**0.45 [0.12, 1.72]**
Total events	13		20			
Heterogeneity: Not applicable						
Test for overall effect: Z = 1.17 (P = 0.24)						
**Total (95% CI)**		**46**		**50**	**100.0%**	**1.71 [0.27, 10.73]**
Total events	34		34			
Heterogeneity: Tau² = 1.78; Chi² = 6.28, df = 2 (P = 0.04); I² = 68%						
Test for overall effect: Z = 0.57 (P = 0.57)						
Test for subgroup differences: Chi² = 3.05, df = 1 (P = 0.08), I² = 67.2%						

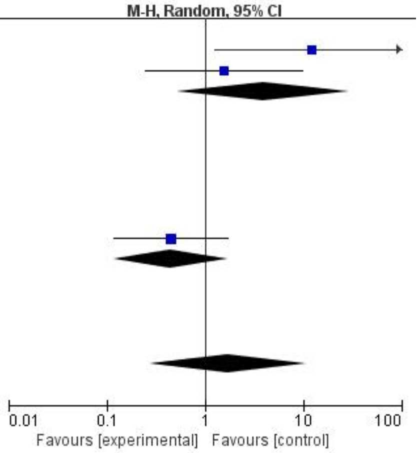

(E) Root lengthening

Minimal instrumentation

Study or Subgroup	Platelet Concentrate Events	Platelet Concentrate Total	BC Events	BC Total	Weight	Odds Ratio M-H, Random, 95% CI
**5.2.1 PRP**						
Jadhav et al. 2012	9	10	6	10	13.7%	6.00 [0.53, 67.65]
Narang et al. 2015	2	5	2	5	13.0%	1.00 [0.08, 12.56]
Shivashanker et al. 2017	5	19	4	15	21.7%	0.98 [0.21, 4.55]
**Subtotal (95% CI)**		**34**		**30**	**48.4%**	**1.49 [0.47, 4.71]**
Total events	16		12			
Heterogeneity: Tau² = 0.00; Chi² = 1.66, df = 2 (P = 0.44); I² = 0%						
Test for overall effect: Z = 0.67 (P = 0.50)						
**5.2.2 PRF**						
Mitta et al. 2019	0	4	1	4	8.2%	0.26 [0.01, 8.52]
Narang et al. 2015	5	5	2	5	8.9%	15.40 [0.56, 425.53]
Sharma et al. 2016	4	4	4	4		Not estimable
Shivashanker et al. 2017	6	20	4	15	22.2%	1.18 [0.27, 5.24]
Uppala et al. 2020	1	8	6	8	12.3%	0.05 [0.00, 0.66]
**Subtotal (95% CI)**		**41**		**36**	**51.6%**	**0.66 [0.08, 5.63]**
Total events	16		17			
Heterogeneity: Tau² = 2.91; Chi² = 8.09, df = 3 (P = 0.04); I² = 63%						
Test for overall effect: Z = 0.38 (P = 0.70)						
**Total (95% CI)**		**75**		**66**	**100.0%**	**1.04 [0.33, 3.25]**
Total events	32		29			
Heterogeneity: Tau² = 0.96; Chi² = 10.44, df = 6 (P = 0.11); I² = 43%						
Test for overall effect: Z = 0.06 (P = 0.95)						
Test for subgroup differences: Chi² = 0.43, df = 1 (P = 0.51), I² = 0%						

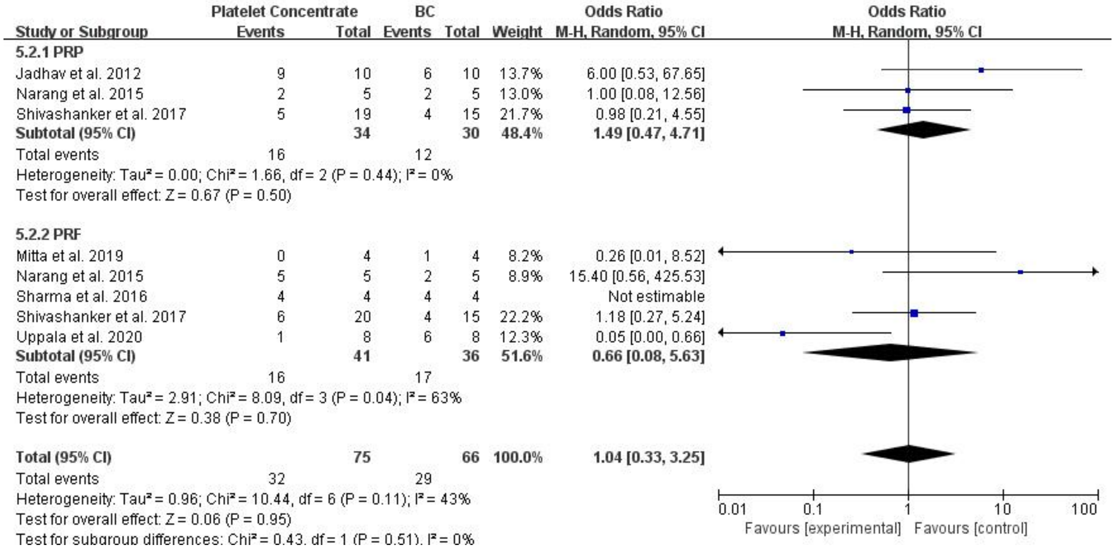

Since fewer than three studies were included, meta-analysis of no instrumentation could not be conducted.

(F) Root canal thickening.

Minimal instrumentation

	Platelet Concentrate		BC			Odds Ratio
**Study or Subgroup**	**Events**	**Total**	**Events**	**Total**	**Weight**	**M-H, Random, 95% CI**
**6.2.1 PRR**						
Jadhav et al. 2012	8	10	3	10	14.6%	9.33 [1.19, 72.99]
Narang et al. 2015	1	5	2	5	8.3%	0.38 [0.02, 6.35]
Shivashanker et al. 2017	5	19	3	15	21.4%	1.43 [0.28, 7.26]
**Subtotal (95% CI)**		**34**		**30**	**44.4%**	**1.99 [0.38, 10.31]**
Total events	14		8			
Heterogeneity: Tau² = 0.96; Chi² = 3.66, df = 2 (P = 0.16); I² = 45%						
Test for overall effect: Z = 0.82 (P = 0.41)						
**6.2.2 PRF**						
Mitta et al. 2019	4	4	3	4	5.6%	3.86 [0.12, 126.73]
Narang et al. 2015	5	5	2	5	6.2%	15.40 [0.56, 425.53]
Sharma et al. 2016	3	4	2	4	7.5%	3.00 [0.15, 59.89]
Shivashanker et al. 2017	6	20	3	15	22.3%	1.71 [0.35, 8.37]
Uppala et al. 2020	2	8	4	8	13.9%	0.33 [0.04, 2.77]
**Subtotal (95% CI)**		**41**		**36**	**55.6%**	**1.68 [0.55, 5.13]**
Total events	20		14			
Heterogeneity: Tau² = 0.13; Chi² = 4.32, df = 4 (P = 0.36); I² = 7%						
Test for overall effect: Z = 0.91 (P = 0.36)						
**Total (95% CI)**		**75**		**66**	**100.0%**	**1.85 [0.79, 4.36]**
Total events	34		22			
Heterogeneity: Tau² = 0.20; Chi² = 8.06, df = 7 (P = 0.33); I² = 13%						
Test for overall effect: Z = 1.41 (P = 0.16)						
Test for subgroup differences: Chi² = 0.03, df = 1 (P = 0.87), I² = 0%						

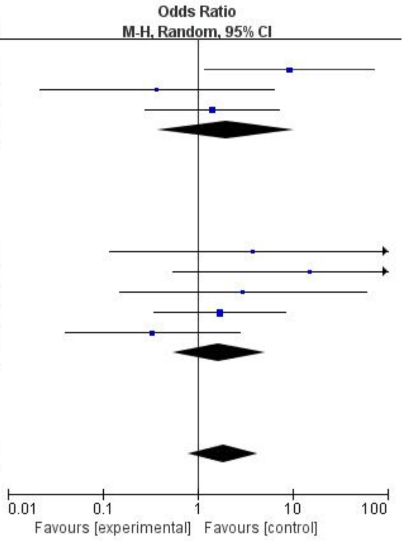

Since fewer than three studies were included, meta-analysis of no instrumentation could not be conducted.
