# Supplementary material for: Are platelet concentrate scaffolds superior to traditional blood clot scaffolds in regeneration therapy of necrotic immature permanent teeth? A systematic review and meta-analysis
Source: BMC Oral Health. 2022 Dec 9;22:589. doi: 10.1186/s12903-022-02605-4 (PMC9733063; doi:10.1186/s12903-022-02605-4)
Supplement: Supplementary file 6 — Additional file 6. The factors of etiology of pulp necrosis. [file 12903_2022_2605_MOESM6_ESM.pdf]

Additional file 6 The factors of etiology of pulp neurosis.

Author	Tooth type	Etiology of pulp necrosis	Groups of Study(n)	included in meta analysis
Bezgin et al. (2015)[14]	Premolars incisors	Caries Trauma	BC (10) PRP (10)	√
Alagl et al. (2017)[16]	Premolars incisors	Caries Trauma	BC (15) PRP (15)	√
Lv et al. (2018)[41]	Premolars Incisors	DE Trauma	BC (5) PRF (5)	√
Meschi et al. (2021)[43]	Premolars Incisors	DE，DI Trauma Caries	BC (18) BC+L-PRF (11)	Data is quantitative analysis and cannot be combined with other studies
Shivashanker et al. (2017)[33]	NR	Caries Trauma	BC(20) PRP(20) PRF(20)	The therapeutic effect was not analyzed according to the etiology
ElSheshtawy et al. (2020)[36]	Incisors	DE Trauma	PRP (14) BC (17)	
Rizk et al. (2020)[40]	Incisors	Caries Trauma	PRP (13) BC+collagen (13)	
Meschi et al. (2021)[43]	Premolars Incisors	DE，DI Trauma Caries	BC (18) BC+L-PRF (11)	
Sharma et al. (2016）[32]	Incisors	Trauma	BC (4) PRF (4) BC+Collagen (4) BC+PLGA (4)	√
Ulusoy et al. (2019)[39]	Incisors	Trauma	BC (22) PRP (22) PRF (22) PP(22)	√
Cheng et al. (2022)[42]	Incisors	Trauma	BC (32) CGF (30)	Data is quantitative analysis and cannot be combined with other studies
Ragab et al. (2019)[35]	Incisors	Trauma	BC(11) BC+PRF (11)	
Ramachandran et al. (2020)[37]	NR	Not reported	BC+PRP+collagen (20) BC (20)	The cause of pulp necrosis was not mentioned.
Uppala et al. (2020)[38]	Incisors	Not reported	PRF(8) BC(8) BC+collagen (8)	
Mittal et al. (2019)	Incisors	Not reported	BC(4) BC+PRF (4)	
Narang et al. (2015)[15]	NR	Not reported	BC (5) PRP+Collagen (5) PRF (5)	
Jadhav et al. (2012)[12]	Incisors	Not reported	BC (10) BC+PRP (10)	
