# Supplementary material for: Are platelet concentrate scaffolds superior to traditional blood clot scaffolds in regeneration therapy of necrotic immature permanent teeth? A systematic review and meta-analysis
Source: BMC Oral Health. 2022 Dec 9;22:589. doi: 10.1186/s12903-022-02605-4 (PMC9733063; doi:10.1186/s12903-022-02605-4)
Supplement: Supplementary file 7 — Additional file 7. The study was divided into two groups for meta analysis according to whether EDTA was used. [file 12903_2022_2605_MOESM7_ESM.pdf]

Additional file 7 The study was divided into two groups for meta analysis according to whether EDTA was used.

Author	Groups of Study(n)	Irrigation	included in meta analysis
Jadhav et al. (2012)[12]	BC (10)<br>BC+PRP (10)	2.5% NaOCl	√
Narang et al. (2015)[15]	BC (5)<br>PRP+Collagen (5)<br>PRF (5)	2.5% NaOCl	√
Sharma et al.<br>(2016) [32]	BC (4)<br>PRF (4)<br>BC+Collagen (4)<br>BC+PLGA (4)	2.5% NaOCl	√
Shivashanker et al. (2017)[33]	BC(20)<br>PRP(20)<br>PRF(20)	5.25% NaOCl	√
Mittal et al. (2019)	BC(4)<br>BC+PRF (4)	2.5% NaOCl	√
Ragab et al. (2019)[35]	BC(11)<br>BC+PRF (11)	5% NaOCl<br>saline.	Data is quantitative analysis and cannot be combined with other studies
Alagl et al. (2017)[16]	BC (15)<br>PRP (15)	1.5% NaOCl<br>2.5% NaOCl<br>0.12% CHX	√
Lv et al. (2018)[41]	BC (5)<br>PRF (5)	1% NaOCl<br>Saline<br>17% EDTA	√
Bezgin et al. (2015)[14]	BC (10)<br>PRP (10)	2.5% NaOCl<br>Saline<br>0.12% CHX<br>5% EDTA	√
Ulusoy et al. (2019)[39]	BC (22)<br>PRP (22)<br>PRF (22)<br>PP(22)	1.25% NaOCl<br>2% CHX<br>17% EDTA.	√
ElSheshtawy et al. (2020)[36]	PRP (14)<br>BC (17)	5.25% NaOCl<br>17% EDTA	
Rizk et al. (2020)[40]	PRP (13)<br>BC+collagen (13)	2% NaOCl<br>17% EDTA	
Ramachandran et al. (2020)[37]	BC+PRP+collagen (20)<br>BC (20)	5.25% NaOCl<br>1% NaOCl<br>sterile water<br>17% EDTA	Data is quantitative analysis and cannot be combined with other studies
Cheng et al. (2022)[42]	BC (32)<br>CGF (30)	0.5-1.5 % NaOCl<br>saline<br>17% EDTA	
Meschi et al. (2021)[43]	BC (18)<br>BC+L-PRF (11)	1.5% NaOCl<br>Saline<br>17% EDTA	
Uppala et al. (2020)[38]	PRF(8)<br>BC(8)<br>BC+collagen (8)	NR	

Four studies were included in the EDTA group[Alagl et al. (2017)[16], Lv et al. (2018)[41], Bezgin et al. (2015)[14, Ulusoy et al. (2019)[39]], and only one study was included in the PRF treatment subgroup[Ulusoy et al. (2019)[39]], so the meta-analysis could not be conducted.
