# Supplementary material for: Are platelet concentrate scaffolds superior to traditional blood clot scaffolds in regeneration therapy of necrotic immature permanent teeth? A systematic review and meta-analysis
Source: BMC Oral Health. 2022 Dec 9;22:589. doi: 10.1186/s12903-022-02605-4 (PMC9733063; doi:10.1186/s12903-022-02605-4)
Supplement: Supplementary file 8 — Additional file 8. The Bgger's and Egg's test of included articals. [file 12903_2022_2605_MOESM8_ESM.pdf]

Additional file 8 The Bgger’s and Egg’s test of included articals.

	Bgger’s		Egg’s test	
	T au	P	t	P
Clinical success	0.000	1.000	1.000	0.374
Response to cold and electric pulp tests	0.095	0.764	1.841	0.125
Periapical healing	0.127	0.586	1.692	0.125
Apex closure	-0.076	0.732	0.364	0.723
Root lengthening	-0.464	0.108	1.775	0.126
Root canal thickening	-0.195	0.474	0.849	0.420
