# Supplementary material for: Are platelet concentrate scaffolds superior to traditional blood clot scaffolds in regeneration therapy of necrotic immature permanent teeth? A systematic review and meta-analysis
Source: BMC Oral Health. 2022 Dec 9;22:589. doi: 10.1186/s12903-022-02605-4 (PMC9733063; doi:10.1186/s12903-022-02605-4)
Supplement: Supplementary file 9 — Additional file 9. The sensitivity analysis of included articals. [file 12903_2022_2605_MOESM9_ESM.pdf]

Additional file 9 The sensitivity analyses of included articals.

(A) Clinical success.

Meta Analysis

Study name	Subgroup within study	Point	Z-Value	Lower limit	Upper limit	p-Value
Shivashanker et al. 2017	PRF	1.020	0.377	0.922	1.128	0.706
Ulusoy et al. 2019	PRF	1.005	0.091	0.909	1.110	0.927
Ulusoy et al. 2019	PRP	0.973	-0.510	0.875	1.082	0.610
		1.000	-0.007	0.920	1.087	0.995

Statistics with study removed; Risk ratio (95% CI) with study removed

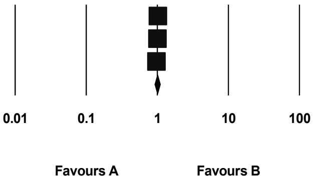

Meta Analysis

(B) Response to cold and electric pulp tests

Meta Analysis

Study name	Subgroup within study	Point	Lower limit	Upper limit	Z-Value	p-Value
Alag et al. 2017	PRP	0.898	0.755	1.069	-1.211	0.226
Bezgin et al. 2015	PRP	1.049	0.753	1.462	0.284	0.776
Shivashanker et al. 2017	PRF	1.128	0.783	1.626	0.647	0.517
Shivashanker et al. 2017	PRP	1.125	0.781	1.621	0.634	0.526
Ulusoy et al. 2019	PRF	1.409	0.761	2.608	1.091	0.275
Ulusoy et al. 2019	PRP	1.420	0.858	2.351	1.363	0.173
Lv et al. 2018	PRF	1.070	0.765	1.498	0.396	0.692
		1.109	0.791	1.554	0.598	0.550

Statistics with study removed; Risk ratio (95% CI) with study removed

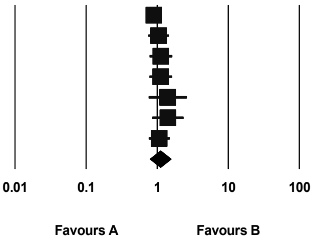

Meta Analysis

(C) Periapical healing

Meta Analysis

Study name	Subgroup within study	Point	Lower limit	Upper limit	Z-Value	p-Value
Bezgin et al. 2015	PRR	1.063	0.976	1.157	1.400	0.162
Jadhav et al. 2012	PRP	1.059	0.974	1.151	1.336	0.181
Narang et al. 2015	PRF	1.060	0.976	1.151	1.377	0.168
Narang et al. 2015	PRP	1.063	0.979	1.155	1.451	0.147
Shivashanker et al. 2017	PRF	1.073	0.986	1.168	1.635	0.102
Shivashanker et al. 2017	PRP	1.048	0.961	1.143	1.066	0.286
Ulusoy et al. 2019	PRF	1.099	0.997	1.212	1.890	0.059
Ulusoy et al. 2019	PRP	1.078	0.972	1.195	1.414	0.157
Uppala et al. 2020	PRF	1.057	0.972	1.150	1.305	0.192
Sharma et al. 2016	PRF	1.062	0.978	1.154	1.424	0.155
Mitta et al. 2019	PRF	1.071	0.986	1.164	1.621	0.105
		1.065	0.981	1.157	1.510	0.131

Statistics with study removed; Risk ratio (95% CI) with study removed

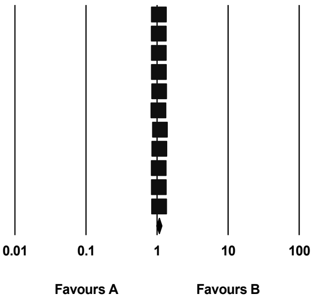

Meta Analysis

(D) Apex closure.

Meta Analysis

Study name	Subgroup within study	Statistics with study removed				
		Point	Lower limit	Upper limit	Z-Value	p-Value
Alag et al. 2017	PRP	0.998	0.804	1.239	-0.019	0.985
Bezgin et al. 2015	PRP	1.086	0.882	1.337	0.774	0.439
Jadhav et al. 2012	PRP	1.022	0.829	1.261	0.204	0.838
Narang et al. 2015	PRF	1.106	0.905	1.352	0.986	0.324
Narang et al. 2015	PRP	1.097	0.896	1.343	0.897	0.370
Shivashanker et al. 2017	PRF	1.114	0.911	1.362	1.051	0.293
Shivashanker et al. 2017	PRP	1.112	0.909	1.360	1.036	0.300
Ulusoy et al. 2019	PRF	1.166	0.942	1.445	1.409	0.159
Ulusoy et al. 2019	PRP	1.167	0.940	1.449	1.397	0.162
Lv et al. 2018	PRF	1.104	0.896	1.361	0.928	0.353
Mitta et al. 2019	PRF	1.079	0.884	1.318	0.751	0.453
Sharma et al. 2016	PRF	1.075	0.873	1.323	0.677	0.498
		1.093	0.897	1.333	0.880	0.379

Risk ratio (95% CI) with study removed

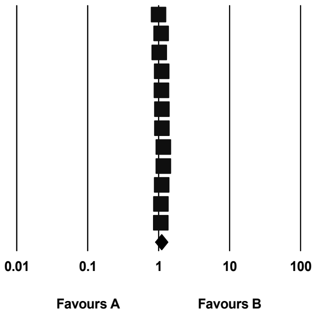

Meta Analysis

(E) Root lengthening

Meta Analysis

Study name	Subgroup within study	Statistics with study removed				
		Point	Lower limit	Upper limit	Z-Value	p-Value
Jadhav et al. 2012	PRP	1.047	0.699	1.568	0.224	0.823
Narang et al. 2015	PRF	1.101	0.781	1.554	0.549	0.583
Narang et al. 2015	PRP	1.199	0.860	1.671	1.068	0.285
Shivashanker et al. 2017	PRF	1.195	0.850	1.680	1.027	0.305
Shivashanker et al. 2017	PRP	1.209	0.861	1.697	1.097	0.273
Lv et al. 2018	PRF	1.269	0.867	1.858	1.225	0.221
Mitta et al. 2019	PRF	1.207	0.871	1.674	1.131	0.258
Uppala et al. 2020	PRF	1.263	0.908	1.756	1.388	0.165
		1.189	0.859	1.645	1.044	0.297

Risk ratio (95% CI) with study removed

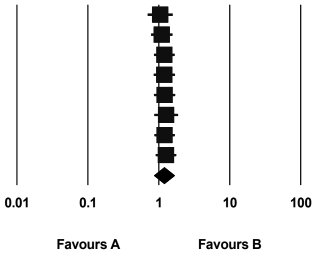

Meta Analysis

(F)

Meta Analysis

Study name	Subgroup within study	Statistics with study removed				
		Point	Lower limit	Upper limit	Z-Value	p-Value
Bezgin et al. 2015	PRP	1.364	0.943	1.974	1.647	0.099
Jadhav et al. 2012	PRR	1.209	0.862	1.698	1.099	0.272
Narang et al. 2015	PRF	1.345	0.972	1.862	1.787	0.074
Narang et al. 2015	PRP	1.345	0.972	1.862	1.787	0.074
Shivashanker et al. 2017	PRF	1.300	0.932	1.813	1.544	0.123
Shivashanker et al. 2017	PRP	1.313	0.942	1.830	1.606	0.108
Lv et al. 2018	PRF	1.364	0.943	1.974	1.647	0.099
Mitta et al. 2019	PRF	1.209	0.862	1.698	1.099	0.272
Sharma et al. 2016	PRF	1.313	0.942	1.830	1.606	0.108
Uppala et al. 2020	PRF	1.387	0.997	1.929	1.942	0.052
		1.313	0.952	1.810	1.662	0.097

Risk ratio (95% CI) with study removed

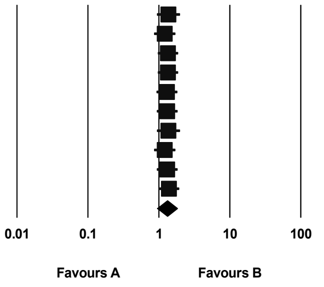

Meta Analysis
